# Benchmarking Large Language Models on the Taiwan Neurology Board Examinations (2018–2024): A Comparative Evaluation of GPT-4o, GPT-o1, DeepSeek-V3, and DeepSeek-R1

**DOI:** 10.3390/bioengineering13030302

**Published:** 2026-03-05

**Authors:** Shih-Yi Lin, Ying-Yu Hsu, Pei-Chun Yeh, Chien-Sheng Hsu, Wu-Huei Hsu, Shih-Sheng Chang, Chia-Hung Kao

**Affiliations:** 1 Graduate Institute of Biomedical Sciences, College of Medicine, China Medical University, Taichung 404, Taiwan; 011833@tool.caaumed.org.tw (S.-Y.L.); 010325@tool.caaumed.org.tw (W.-H.H.); 2 Division of Nephrology and Kidney Institute, China Medical University Hospital, Taichung 404, Taiwan; 3 National Changhua Senior High School, Changhua 500, Taiwan; yingyu961120@gmail.com; 4 Artificial Intelligence and Robotics Innovation Center, China Medical University Hospital, Taichung 404, Taiwan; 008252@tool.caaumed.org.tw (P.-C.Y.); 011996@tool.caaumed.org.tw (S.-S.C.); 5 Department of Neurology, China Medical University Hospital, Taichung 404, Taiwan; 029986@tool.caaumed.org.tw; 6 Department of Chest Medicine, China Medical University Hospital, Taichung 404, Taiwan; 7 Division of Cardiovascular Medicine, Department of Medicine and School of Medicine, China Medical University, Taichung 110, Taiwan; 8 Department of Nuclear Medicine and PET Center, China Medical University Hospital, Taichung 404, Taiwan; 9 Department of Bioinformatics and Medical Engineering, Asia University, Taichung 404, Taiwan

**Keywords:** large language models (LLMs), neurology board examination, GPT-4o/GPT-o1, DeepSeek-R1/DeepSeek-V3

## Abstract

**Background and Purpose:** Neurology requires integration of clinical reasoning, imaging interpretation, and current knowledge, making it an ideal field for evaluating large language models (LLMs). **Methods:** Using 1715 questions from the Taiwan Neurology Board Examination (2018–2024), we assessed four LLMs—GPT-4o, GPT-o1, DeepSeek-V3, and DeepSeek-R1—across four formats: single-choice, multiple-choice, true–false, and image-based items. **Results:** GPT-o1 achieved the highest overall accuracy (83.86%) and demonstrated strong performance on cognitively demanding tasks (82.50% on true–false; 77.26% on image-based). DeepSeek-V3 scored lowest (65.62%) and showed the greatest variability. Statistical analyses confirmed significant inter-model differences (*p* < 0.01). Accuracy declined across all models in 2024, coinciding with shifts in question design. DeepSeek-R1 was further penalized by alignment-based refusals, resulting in up to 3.81% score loss. **Conclusions:** These results position the Taiwan Neurology Board Exam as a rigorous benchmark for LLM evaluation and underscore GPT-o1’s potential utility in neurology education and decision support.

## 1. Introduction

Since the launch of ChatGPT based on GPT-3.5 at the end of 2022, the platform rapidly gained popularity, reaching 100 million users faster than TikTok and Instagram [1]. Subsequently, the GPT series—including GPT-3.5, GPT-4.0, and GPT-4o (formerly referred to as GPT-4o mini)—along with Google’s Gemini series, have been widely adopted and evaluated globally [2]. These large language models have demonstrated broad applicability across diverse domains, including bioengineering, industrial automation, information processing, document generation, creative content production, and medical practice [3,4,5,6,7].

Recent advancements in large language models (LLMs) have transformed the landscape of medical education, decision support, and knowledge retrieval [8,9,10]. Among various specialties, neurology presents a particularly rigorous testbed for evaluating LLM capabilities, as it requires the integration of complex clinical reasoning, multimodal imaging interpretation, and continuously evolving medical knowledge [11]. These demands are compounded by the nuanced symptomatology and heterogeneous diagnostic pathways that characterize neurological disorders. As such, neurology is an ideal domain in which to assess how effectively LLMs can navigate real-world medical scenarios.

The Taiwan Neurology Board Examination offers a structured and well-established framework for this purpose. Unlike standard multiple-choice assessments, it incorporates four distinct question types: A-type (single-best answer), C-type (multiple-correct), K-type (true–false matrix), and A-II-type (image-based, clinically contextualized items) [12]. These formats test not only factual recall but also clinical judgment, reasoning under uncertainty, and the interpretation of neuroimaging—areas where AI models must demonstrate not just linguistic fluency but domain-specific competence.

In this study, we selected four LLMs for evaluation: GPT-4o and GPT-o1 (developed by OpenAI), and DeepSeek-V3 and DeepSeek-R1 (recently released models by DeepSeek) [13,14]. GPT series was chosen based on findings from our previous research involving Taiwan’s medical specialty examinations, where both models consistently achieved top-tier performance [15]. In contrast, DeepSeek-V3 and DeepSeek-R1 were selected due to their recent claims of outperforming current state-of-the-art models, including in tasks requiring multilingual and medical-domain reasoning [16,17,18]. Their inclusion allows us to evaluate whether these newer entrants deliver tangible performance improvements when benchmarked against the demanding and multimodal nature of the Taiwan Neurology Board Exam.

By leveraging seven years of authentic board exam content (2018–2024), this study aims to conduct a longitudinal and format-specific performance comparison of these LLMs, with a focus on identifying strengths, limitations, and potential applications in neurology education and clinical support. Through detailed statistical analysis—including accuracy, variability, and effect sizes—we aim to offer a nuanced assessment of how well current-generation LLMs meet the standards expected in specialty-level neurology certification.

## 2. Methods

### 2.1. Study Design and Dataset

This study conducted a retrospective, quantitative evaluation of four large language models (LLMs) using content from the Taiwan Neurology Board Examination administered between 2018 and 2024. We selected 2018 as the starting year primarily for methodological comparability: from 2018 onward, the examination maintained a stable blueprint with a constant annual total of 245 items and a fixed question-type composition (A/C/K/All = 145/30/40/30), with no B-type (case-set) items (Table S1). In contrast, pre-2018 examinations (2014–2017) differed substantially in both total items (214–230) and format, including large and variable numbers of B-type case-set items (60–159 per year), which would introduce a structural break and confound across-year comparisons/benchmarking. The examination is a nationally standardized, physician-level certification required for board licensure in neurology in Taiwan. Each year, the exam comprises 245 items, yielding a total of 1715 questions over seven years. The questions were categorized into four formats: A-type (single-best-answer), C-type (multiple-correct-answer), K-type (true–false matrix), and A-II-type (image-based single-best-answer). According to the official scoring scheme, A-type and C-type items contribute 1 point per item, whereas K-type and A-II-type items contribute 2 points per item. The examination underwent structural modifications in 2024, including a reduction in the number of C-type questions (from 30 to 20) and an increase in A-II-type questions (from 30 to 40), while maintaining a total of 245 items annually. Because the annual maximum score depends on the item-type composition under the points-per-item scheme (A/C = 1; K/A-II = 2), the 2024 redistribution increased the maximum attainable score from 315 (2018–2023) to 325 points (2024) (Tables S1 and S2).

### 2.2. Model Selection

Four large language models (LLMs) were evaluated in this study: GPT-4o and GPT-o1, both developed by OpenAI, and DeepSeek-V3 and DeepSeek-R1, developed by DeepSeek. These models were chosen to represent both widely established and emerging generative AI systems in the public domain.

GPT-4o, released in May 2024, is OpenAI’s latest flagship multimodal model capable of processing text, images, and audio inputs. It is publicly available via the ChatGPT web interface (https://chat.openai.com/, accessed on 4 February 2025 to 2 March 2025) under the GPT-4 system, replacing the previous GPT-4 model with improved latency and performance.

GPT-o1, introduced by OpenAI in late 2023, is a smaller variant optimized for general-purpose reasoning and speed, also accessible through the ChatGPT platform.

DeepSeek-V3 and DeepSeek-R1 are part of a new generation of open-access LLMs released by DeepSeek, a research group based in China. DeepSeek-V3 was publicly released in March 2024, followed by DeepSeek-R1 in April 2024. Both are accessible through DeepSeek’s official web-based chat interface (https://chat.deepseek.com/, accessed on 4 February 2025 to 2 March 2025) and are positioned as high-performance models for multilingual and technical tasks. The DeepSeek team claims that these models achieve performance competitive with, or superior to, GPT-4 in various academic and reasoning benchmarks.

All models were accessed through their respective official graphical user interfaces (GUIs) to simulate typical user interactions. No API or scripted automation was used, ensuring that model behavior reflected realistic, end-user conditions.

### 2.3. Model Access and Evaluation Procedures

The evaluation of large language models (LLMs) was conducted between 4 February 2025 and 2 March 2025. All four models (GPT-4o, GPT-o1, DeepSeek-V3, and DeepSeek-R1) were evaluated during this same study period via their official online platforms. For text-based questions, models were prompted in batched chat inputs of up to 10 items per message; if fewer than 10 items remained, the batch was adjusted accordingly. For image-based questions, each item was prompted individually to maintain clarity and ensure precise evaluation. This batching difference was adopted due to practical interface constraints and throughput considerations during large-scale evaluation. To mitigate potential within-batch context effects, each item within a batched prompt was clearly numbered and separated, and the model was instructed to answer each item independently without relying on other items in the same batch. Importantly, the unit of evaluation and scoring remained the individual exam item: each item was answered exactly once per model, even when multiple text items were entered within a single chat input. This procedural asymmetry may introduce within-batch context/interference effects for text questions and should be considered when interpreting comparisons between text-only and image-based performance. All questions were manually entered from the official exam materials, without providing any answer keys or solution explanations. For DeepSeek-V3 and DeepSeek-R1, A-II items were provided as full-stem screenshots when applicable to preserve the complete question content under platform constraints (see Supplementary Methods). Prompts did not include external links or references. Model outputs were saved, and scoring was performed offline by the research team using the official answer key, which was never shown to the models. Although these procedures reduce leakage risk, we cannot fully verify provider-side retrieval mechanisms or proprietary training data for each platform; residual leakage risk is therefore acknowledged as a limitation. We did not use prompt reformulation (e.g., explicitly clarifying clinical intent) to resolve refusals, in order to preserve a standardized benchmarking setting; clinically framed re-prompts may be evaluated in future work as a separate interactive-assistant scenario. To accommodate platform-specific access limits and stability constraints, we distributed single-pass item attempts (i.e., one response attempt per exam item) across multiple accounts where needed; operational access/logistics details (e.g., account counts, timing, batching) are provided in the Supplementary Methods. Regardless of access constraints, each exam item (including image-based items) was submitted once per model, and the total number of prompted items was identical across models. Prompting followed a fixed, minimal instruction to preserve comparability across platforms. For each item, we provided the original question stem and answer options verbatim and asked the model to select the single best answer by outputting only the option letter/label, without additional hints, explanations, or external resources. We used the standard chat interface available during the study period and did not instruct the models to browse the web. We did not enable any special research mode (e.g., Deep Research) or tool-assisted browsing workflow. A schematic overview of the evaluation workflow and platform-specific input constraints is provided in Figure S6.

Input strategies were adapted based on the interface capabilities of each model. For GPT-4o and GPT-o1, image-based questions were submitted using text prompts with direct image-file upload. In contrast, in the public interface used for DeepSeek-V3 and DeepSeek-R1 during the study period, uploaded images were handled primarily via extraction of embedded text rather than reliable visual interpretation. When an upload contained little or no readable text (e.g., an image-only medical figure), text extraction could fail, preventing dependable processing (Figures S1–S3). Therefore, for DeepSeek-V3 and DeepSeek-R1, for A-II-type items we uploaded full-stem screenshots that contained the entire question text, associated image, and any formatting (e.g., tables) to ensure that all required textual information was accessible under this constraint. A specific content restriction was observed in DeepSeek-R1, a reasoning-optimized model. When encountering prompts containing region-specific terminology (e.g., “Taiwan”), the model sometimes returned a refusal such as: “Sorry, that’s beyond my current scope. Let’s talk about something else” (Figure S4). Because refusals reflect alignment/policy behavior rather than epistemic error, we categorized such outputs as “refusals” and report their frequency and score impact separately (Table S8; see item-level list in Table S10). Under our standardized single-pass scoring framework, refusal outputs contributed zero points because no actionable answer was provided. This issue did not affect GPT-4o, GPT-o1, or DeepSeek-V3, which processed these prompts without refusal in our evaluation. Each model was prompted with the original question content using standardized, human-readable input. For C-type and K-type items, although each question is conceptually based on multiple statements, examinees select a single option that encodes the required combination; therefore, both C-type and K-type items were scored dichotomously (full credit if the selected option matches the answer key; otherwise zero), rather than using component-level partial credit. All responses were reviewed manually and scored using the official answer key. Item points followed the official scheme (A-type = 1, C-type = 1, K-type = 2, A-II-type = 2): a correct option received full item credit and an incorrect option received zero.

### 2.4. Statistical Analysis

Descriptive statistics were used to summarize model performance across question types and examination years. For each large language model (LLM), we calculated the mean scores, standard deviations, and percentage accuracy across A-type, C-type, K-type, and A-II-type questions. Average performance was analyzed both annually and cumulatively across the 2018–2024 evaluation period. To ensure cross-year comparability under the 2024 scoring-structure change, we additionally reported an annual normalized total score computed as (raw score/annual maximum points) × 100, where the maximum points were 315 for 2018–2023 and 325 for 2024.

Tests for normality were conducted using both the Kolmogorov–Smirnov and Shapiro–Wilk tests. As most datasets deviated significantly from a normal distribution (*p* < 0.001), nonparametric tests were applied for further analysis. The Kruskal–Wallis H test was used to assess overall differences in performance between models within each question type. Where statistically significant differences were detected (*p* < 0.05), pairwise comparisons were conducted using Mann–Whitney U tests with Bonferroni correction (adjusted α′ = 0.0083) to control for multiple comparisons.

Effect sizes (r) were calculated for pairwise comparisons to evaluate the magnitude of observed differences, and interpreted according to conventional thresholds (small ≥ 0.1, moderate ≥ 0.3, large ≥ 0.5). Mean rank data were used to assess relative performance positioning in each model-question type combination.

All statistical analyses were performed using SPSS (version 26.0; IBM Corp., Armonk, NY, USA) or equivalent statistical software. A two-tailed *p*-value < 0.05 was considered statistically significant unless otherwise corrected.

This work has been reported in line with the STROCSS criteria [19].

## 3. Results

Table S1 outlines the structural composition of the Taiwan Neurology Board Examination from 2018 to 2024. Each year, the exam consisted of 245 questions, divided into Part I (A-type) and Part II (C-type, K-type, and A-II-type). While the overall format remained stable, 2024 introduced a key structural change: the number of C-type questions was reduced from 30 to 20, and A-II-type questions increased from 30 to 40. Despite the unchanged total number of questions, the maximum possible score increased from 315 to 325 points in 2024. This increase was attributable to the points-per-item scheme (A- and C-type = 1 point; K- and A-II-type = 2 points) coupled with the 2024 redistribution toward more A-II-type items (Tables S1 and S2). Changes in score distribution are presented in Table S2, which shows that from 2018 to 2023, A-type questions consistently contributed the largest share of total points (46.03%), followed by K-type (25.40%), A-II-type (19.05%), and C-type (9.52%). In 2024, this distribution shifted: A-type dropped slightly to 44.62%, C-type decreased notably to 6.15%, and A-II-type rose to 24.62%, matching K-type. These adjustments mirror the revised composition of question types and suggest a greater emphasis on applied and visual interpretation skills.

Model-level performance across question types and years is summarized in Table S3. Overall, GPT-o1 demonstrated the strongest performance across formats and years, with its advantage most evident in A-type (single-best-answer) and K-type (logic/reasoning) questions. Across models, performance patterns were broadly stable by format, whereas 2024 showed a general decline consistent with the exam’s structural shift and increased emphasis on image/table-only content (see Tables S1 and S7 for composition and era comparison). Detailed year-by-year correct counts are provided in Table S3.

As summarized in Table S4, normalized performance confirmed GPT-o1’s consistent superiority across all years, with scores ranging from 85.1% to 89.5%, and peak performance observed in 2018 and 2019 (both 89.5%). Because item formats differ in item-point ranges (A/C = 1 vs. K/A-II = 2), the normalized annual total score (% of annual maximum) provides a fairer basis for cross-format comparison than directly comparing raw scores across heterogeneous point scales. In contrast, DeepSeek-V3 had the lowest normalized scores, ranging from 61.3% to 67.5%, with its lowest in 2024. GPT-4o and DeepSeek-R1 showed more moderate and stable performance, with normalized scores between 67.2% and 72.6%. Normalized scores (Table S4) and accuracy by format (Table S5) consistently ranked GPT-o1 highest overall, followed by DeepSeek-R1 and GPT-4o, with DeepSeek-V3 lowest. Across models, A-type items were answered most reliably, whereas K-type items were consistently the most challenging, indicating that logic-intensive questions provide stronger discrimination among models (Tables S5 and S6). Full format-specific accuracies are reported in Table S5.

Patterns of relative question difficulty were analyzed in Table S6. A-type questions were the easiest overall (mean accuracy: 83.70%), followed by C-type (72.52%) and A-II-type (69.16%), while K-type was clearly the most difficult (65.36%). GPT-o1 led in every format, demonstrating both accuracy and adaptability across cognitive domains.

The two-era comparison (2018–2023 vs. 2024) is presented in Table S7 and shows a consistent decline in accuracy across models in 2024. Importantly, despite the across-the-board decrease, the relative difficulty ordering by format was unchanged, with K-type remaining the most challenging category. Detailed percentages by format and era are provided in Table S7. Table S8 explores the presence of restricted keyword content (e.g., “Taiwan”) and its impact on model performance. Across the years, between 3 and 9 such questions appeared annually, with the highest counts observed in 2019 and 2024. DeepSeek-R1 consistently refused to respond to these items, resulting in zero points and annual losses ranging from 1.27% to 3.81% of the total possible score. GPT-o1, GPT-4o, and DeepSeek-V3 handled these questions without issue, maintaining full engagement with keyword-containing content.

Statistical consistency was assessed via descriptive metrics in Table 1, where GPT-o1 had the highest mean scores across all question types: A-type (0.91 ± 0.29), C-type (0.83 ± 0.38), K-type (1.65 ± 0.76), and A-II-type (1.55 ± 0.84). In contrast, DeepSeek-V3 and GPT-4o showed lower means and greater standard deviation, particularly for K-type questions (SD = 1.00), indicating greater instability in logic-based performance. A-type items showed the least variability across models, with SDs ranging from 0.29 to 0.41.

Normality tests presented in Table 2 revealed that score distributions for all models and question types significantly deviated from normality (Kolmogorov–Smirnov and Shapiro–Wilk, *p* < 0.001). This justified the use of non-parametric statistical methods for model comparison. Importantly, because our formal inter-model comparisons (Table 3, Table 4 and Table 5) are rank-based (Kruskal–Wallis and Mann–Whitney U tests), the statistical inference remains robust even when question types differ in scoring ranges (0–1 vs. 0–2 points).

Table 3 shows that GPT-o1 achieved the highest Kruskal–Wallis mean ranks across all question types, including A-type (2182), C-type (443.5), K-type (656.5), and A-II-type (476.5). DeepSeek-V3 consistently ranked lowest in all categories, reinforcing its overall weaker performance.

Results of the Kruskal–Wallis H test are summarized in Table 4, revealing significant performance differences across all question types: A-type (H = 58.923, *p* < 0.001), C-type (H = 15.966, *p* = 0.001), K-type (H = 67.073, *p* < 0.001), and A-II-type (H = 12.416, *p* = 0.006). These findings confirm that model performance varied meaningfully across formats.

To identify which specific model pairs differed, pairwise comparisons using the Mann–Whitney U test were conducted (Table 5). After Bonferroni correction (α′ = 0.0083), GPT-o1 significantly outperformed GPT-4o and DeepSeek-V3 in both A-type and K-type questions (*p* < 0.001), and also outperformed GPT-4o in C-type (*p* = 0.006). No significant differences were found in A-II-type comparisons after correction, suggesting more convergent performance in this domain.

Table 6 reports nonparametric effect sizes (r) for these comparisons. The strongest effect was found in K-type questions between GPT-o1 and GPT-4o (r = 0.293, moderate). Most other comparisons showed small or negligible effects; for example, A-type comparisons yielded r-values between 0.022 and 0.169, while A-II-type items consistently showed effects below r = 0.10, reinforcing their limited discriminatory power among models.

To supplement the main statistical findings, Table S9 provides the complete results of pairwise comparisons between the four large language models (GPT-4o, GPT-o1, DeepSeek-V3, and DeepSeek-R1) using the Mann–Whitney U test across all question types. These unadjusted analyses offer a detailed view of model-to-model differences in performance distribution, supporting the identification of significant contrasts—particularly in A-type and K-type items. Table S10 documents all instances where DeepSeek-R1 refused to answer questions containing the keyword “Taiwan.” These refusals, which occurred across multiple exam years and question formats, underscore a model-specific content restriction not observed in the other LLMs, highlighting a critical limitation in DeepSeek-R1’s applicability to Taiwan-based clinical and educational content.

## 4. Discussion

This study revealed statistically detectable differences in performance among large language models when evaluated on a comprehensive, multi-format medical board examination in neurology. GPT-o1 showed a consistent advantage over the other models across examination years and several question formats; however, the magnitude of many pairwise differences was small (Table 6), and practical impact should therefore be interpreted cautiously. These results suggest that GPT-o1 may offer modest gains in generalization and reasoning under this evaluation setting rather than uniformly large improvements. Statistically significant differences were observed across models, but these should be considered alongside effect sizes to avoid overstating practical relevance. Throughout the manuscript, we deliberately focus on methodological evaluation and educational implications, and avoid sociopolitical commentary to maintain academic objectivity.

In contrast, DeepSeek-R1 produced alignment-based refusals on certain prompts, which resulted in lost scoring opportunities under our single-pass scoring framework. Because refusals reflect policy/alignment constraints rather than cognitive error, we report refusals separately from incorrect answers and interpret their impact primarily as a usability limitation in this evaluation setting (Figure S4; Tables S8 and S10). More broadly, these findings underscore the importance of documenting evaluation eras: changes in testing interfaces, access controls, model deployments, or exam structure can meaningfully affect reproducibility and comparability over time. Accordingly, future benchmarking should pre-specify prompts, record model/version metadata and run settings, and preserve evaluation artifacts to enable robust longitudinal comparisons. In addition, while prompt reformulation may help disambiguate clinical intent from geopolitical interpretation in refusal-prone cases, we did not employ iterative reformulation in this study to preserve comparability across models; we instead note this as a methodological constraint and a direction for future work. Additionally, a general decline in performance was noted in the final year of analysis, which coincided with structural changes to the exam—namely, a reduction in traditional multiple-choice items and an increased emphasis on image-based content. This trend highlights the influence of question format on LLM adaptability. Moreover, logic-based question types proved especially useful for differentiating between models, reinforcing their value as a benchmark for assessing reasoning performance. Together, these findings provide important insight into the readiness of current LLMs for integration into neurology education and AI-assisted assessment systems, while also identifying format-specific limitations that warrant further investigation. Importantly, board-style exam performance reflects accuracy within a controlled testing format and should not be conflated with real-world clinical reliability or safety. In neurology training, such models may be most useful for educational support—such as generating explanations for practice questions, providing formative feedback, and helping learners identify knowledge gaps—where outputs can be reviewed and corrected by instructors. By contrast, clinical decision support demands additional safeguards (e.g., domain grounding to patient-specific evidence, auditability, and human oversight), and benchmark gains in exam accuracy alone are insufficient to justify unsupervised clinical use.

While we further examined model performance through pairwise comparisons, differences emerged that highlight relative strengths and limitations across question formats. GPT-o1 was statistically better than GPT-4o and DeepSeek-V3 in A-type and K-type questions and also outperformed GPT-4o in C-type questions; however, most corresponding effect sizes were small (Table 6), indicating that these advantages are generally modest in magnitude. Accordingly, these findings suggest that GPT-o1 provides consistent but limited gains in factual recall and logical judgment under this benchmark, rather than a uniformly large practical advantage [20]. Relative to prior LLM benchmarking studies on other medical examinations and test formats, our findings align with commonly reported patterns: performance is typically stronger on text-based multiple-choice items than on image-centric or visually grounded reasoning tasks, and results can be sensitive to evaluation protocols (e.g., one-shot vs. interactive prompting), language and region-specific content, and platform/interface constraints. Taken together, the emerging consensus is that board-exam benchmarks are most informative for assessing controlled-format competence and potential educational utility, whereas clinical reliability and safety cannot be inferred from exam accuracy alone and require additional safeguards (e.g., evidence grounding, auditability, and human oversight). In practical terms, such modest gaps may translate into only a small number of additional correctly answered items per exam and are most relevant for educational support (e.g., exam preparation or formative feedback), rather than as a stand-alone basis for clinical decisions without human oversight. Interestingly, no significant differences were found among any models for A-II-type (image-based) questions. This suggests a convergence of performance in visual interpretation tasks, which may reflect either a shared limitation in current LLMs’ ability to process image-contextual reasoning or a possible ceiling effect imposed by the structure of the image-based questions themselves [21]. However, because image ingestion was not identical across platforms (direct image upload vs. screenshot-based input), this interpretation should be considered conditional on the tested interfaces and input modalities. This interpretation is supported by recent findings showing that even advanced multimodal large language models (MLLMs) often struggle with basic visual reasoning tasks—such as counting, object localization, and spatial inference—despite their strong language capabilities. These limitations suggest that current MLLMs may have inherent weaknesses in processing and interpreting visual information, which aligns with the observed performance plateau across all models in our image-based question set [21].

In practice, this indicates that while LLMs may be useful for assisting with knowledge-based or logic-driven tasks, image-centered diagnostic reasoning still presents a major bottleneck for current-generation models and should not be delegated without human oversight. This concern is reinforced by recent findings showing that state-of-the-art large multimodal models (LMMs)—including GPT-4o, GPT-4V, and Gemini Pro—can perform worse than random guessing when confronted with specialized diagnostic questions in medical visual question answering (Med-VQA). These results highlight significant limitations in the ability of current models to interpret fine-grained, clinically relevant visual cues, suggesting that such tasks remain beyond the scope of reliable AI automation in high-stakes medical environments [22].

The observed differences between GPT-4o and both DeepSeek-V3 and DeepSeek-R1 further emphasize the performance gradient across model tiers. GPT-4o consistently outperformed both DeepSeek models in multiple question formats, particularly in A-type (factual recall) and K-type (logic-based) questions—domains that demand precise knowledge, clear reasoning, and consistent response patterns. Although DeepSeek models represent promising and accessible alternatives, they demonstrated weaker reliability in tasks requiring factual accuracy and deductive logic. This finding underscores the need for caution when deploying emerging LLMs in educational or clinical contexts, where misinterpretation could carry significant consequences.

Supporting this concern, a recent study evaluating DeepSeek R1’s medical reasoning across 100 MedQA clinical cases found that, despite achieving relatively high diagnostic accuracy, the model exhibited notable reasoning limitations [23]. These included anchoring bias, difficulty reconciling conflicting information, shallow exploration of differential diagnoses, knowledge gaps, and premature prioritization of definitive treatments over intermediate care [23]. Such findings highlight the specific cognitive vulnerabilities that can arise with DeepSeek models in high-stakes medical settings and reinforce concerns about their readiness for complex clinical decision-making. Overall, these results emphasize the importance of context-specific benchmarking. Model claims of general superiority may not translate across specialized domains like neurology, where reasoning depth, domain fluency, and interpretive nuance are critical to performance and safety.

The performance comparisons between GPT-o1 and DeepSeek-V3, as well as GPT-o1 and DeepSeek-R1, further underscore the differences in model maturity, design philosophy, and optimization focus between OpenAI’s and DeepSeek’s large language models. GPT-o1—a streamlined and cost-efficient variant of GPT-4 developed by OpenAI—demonstrated statistically and practically significant superiority over both DeepSeek models in formats requiring factual precision (A-type) and logical consistency (K-type). These findings suggest that OpenAI’s models remain more refined in handling structured, high-stakes content, likely owing to training on larger, higher-quality datasets and more extensive instruction tuning. Supporting this, DeepSeek-R1, a reasoning-focused model trained using reinforcement learning techniques, has been shown to perform well in mathematical and logic-based benchmarks [24]. However, studies report that it still performs slightly below GPT-o1 on standardized evaluations such as the MMLU, particularly in tasks requiring factual accuracy [24]. Additionally, when evaluated in complex bilingual ophthalmology scenarios, DeepSeek-R1 demonstrated strengths in some reasoning tasks but showed inconsistent performance relative to OpenAI models [25]. These results reflect how differences in design priorities and optimization strategies—such as multilingual fine-tuning versus factual depth—can produce varied capabilities across domains, further reinforcing GPT-o1’s robustness in high-cognitive-load medical assessments.

In contrast, DeepSeek-V3 and DeepSeek-R1—while positioned as high-performing, open-access alternatives—exhibited lower reliability and greater variability in their responses. This may reflect differences in model scale, alignment strategies, and multilingual fine-tuning. Although DeepSeek-R1 is advertised as a “reasoning-optimized” model, its performance did not surpass GPT-o1 in logic-based tasks [24], and was further limited by alignment-based refusals on region-specific terms, indicating areas where model policy or alignment constraints may inhibit its utility in medical applications [23]. These results underscore the importance of evaluating models not only on general benchmark scores but also on contextual usability and transparency in medical practice. From a service perspective, both GPT-o1 and GPT-4o benefit from stable infrastructure, frequent updates, and long-term community feedback, which likely contribute to their more consistent and polished behavior across complex task types [26]. While DeepSeek’s offerings are valuable as free, open-access models [27], the observed performance gap suggests they are not yet equivalent replacements for OpenAI’s models in specialized domains such as neurology board-level assessment.

Despite the strengths of using real-world board examination content and multiple cognitive domains to evaluate model performance, this study has several important limitations. First, all models were accessed through their respective official web-based graphical user interfaces, which are influenced by external factors such as server load, latency, and session variability. Although care was taken to standardize input conditions, subtle differences in user experience or rendering of visual content could have influenced responses. Second, the evaluation relied on a single interaction per question, which does not reflect the iterative nature of human-AI engagement, where clarifications or follow-up prompts might lead to improved answers. Third, while the models were presented with identical content, platform-specific differences in image handling required alternative input methods—such as uploading screenshots for DeepSeek models—which may have impacted interpretation quality despite our best efforts to preserve information fidelity. Accordingly, differences observed on A-II-type (image-based) items may reflect both intrinsic model capability and input-pipeline fidelity/modality support; therefore, cross-model comparisons on image-based performance should be interpreted with caution. Fourth, some text-based items were submitted in small batches depending on interface constraints, which could introduce minor context effects, although prompts were designed to minimize cross-item leakage. Fifth, although we implemented safeguards to reduce information leakage (manual entry, no answer keys provided, offline scoring), we cannot fully verify provider-side retrieval mechanisms or proprietary training data; residual leakage risk therefore remains. Additionally, DeepSeek-R1’s refusal to respond to questions containing the term “Taiwan” highlights the challenge of evaluating models with undisclosed or restrictive alignment filters that are beyond the control of the evaluator. Finally, while the Taiwan Neurology Board Examination provides a robust benchmark for specialty-level knowledge and reasoning, the findings may not fully generalize to other domains of medicine, different languages, or clinical environments. These limitations underscore the need for ongoing refinement in both model development and evaluation frameworks to ensure consistent, equitable, and interpretable assessments across varied medical contexts.

The consistent refusal of DeepSeek-R1 to engage with questions containing the term “Taiwan” highlights a broader concern regarding hidden or undocumented alignment restrictions [28]. While this keyword-triggered refusal was clearly identifiable due to its recurrence and relevance in Taiwan-specific content, it is plausible that other sensitive or geo-political terms may also trigger similar behavior, albeit less visibly. These unnoticed or undocumented keyword filters may silently suppress model responses or produce generic disclaimers, reducing transparency and reliability—especially in domains like medicine, where regional terminology, drug names, or institutional references are often essential.

In clinical or educational applications, such opaque content moderation presents a significant risk of partial or misleading output, particularly if end users are unaware that the model has actively avoided addressing part of the input. In the context of Taiwan’s medical system and neurological disease landscape, which often includes region-specific terminology, such behavior could undermine the model’s utility. These findings highlight the importance of thorough regional benchmarking, as well as the need for LLM developers to disclose alignment policies and refusal triggers, especially when models are deployed in high-stakes or context-sensitive environments.

Future research should explore model performance using multimodal input pipelines that more closely replicate clinical environments, including conversational or follow-up query interactions. Integrating real-time reasoning chains, visual explanation outputs, or structured prompt engineering could further clarify how models arrive at their conclusions. Additionally, collaboration with platform developers to ensure transparency in content filtering and alignment policies will be essential for fair benchmarking, especially in high-stakes educational or regional settings. Future research should explore model performance using multimodal input pipelines that more closely replicate clinical environments, including conversational or follow-up query interactions. Integrating real-time reasoning chains, visual explanation outputs, or structured prompt engineering could further clarify how models arrive at their conclusions. Additionally, collaboration with platform developers to ensure transparency in content filtering and alignment policies will be essential for fair benchmarking, especially in high-stakes educational or regional settings. Future studies should also (i) adopt more standardized multimodal ingestion across platforms to ensure comparable handling of images and tables; (ii) evaluate interactive prompting settings, including structured re-prompts and clinically framed clarifications for refusal-prone items, as a distinct assistant-use scenario; (iii) document evaluation-era metadata (platform/interface, run conditions, and model/version information) to support reproducible longitudinal comparisons; and (iv) extend benchmarking to additional years, specialties, languages, and model families to test generalizability and clinical relevance. Expanding evaluations to include other medical specialties, languages, and question formats will help validate the generalizability of findings and refine the criteria by which medical LLMs are assessed.

## 5. Conclusions

This study demonstrates that current-generation LLMs, particularly GPT-o1, are capable of engaging with complex, multimodal, and high-level neurology board examination content. While performance varies across models and question formats, the results affirm the potential of LLMs to support medical education and specialty training. However, interface constraints, content limitations, and question structure continue to shape model behavior. Ongoing evaluation and model refinement will be critical to ensuring that these tools are both reliable and responsibly integrated into clinical and educational practice.

## Figures and Tables

**Table 1:** Comparison of Mean Scores and Standard Deviations by Question Type and Large Language Model.

Type	Model	Mean ± SD
A-type	GPT_4o	0.83 ± 0.38
	GPT_o1	0.91 ± 0.29
	DeepSeek-V3	0.80 ± 0.40
	DeepSeek_R1	0.81 ± 0.40
C-type	GPT_4o	0.69 ± 0.47
	GPT_o1	0.83 ± 0.38
	DeepSeek-V3	0.67 ± 0.47
	DeepSeek_R1	0.71 ± 0.46
K-type	GPT_4o	1.11 ± 1.00
	GPT_o1	1.65 ± 0.76
	DeepSeek-V3	1.08 ± 1.00
	DeepSeek_R1	1.39 ± 0.92
A-II	GPT_4o	1.38 ± 0.93
	GPT_o1	1.55 ± 0.84
	DeepSeek-V3	1.24 ± 0.97
	DeepSeek_R1	1.36 ± 0.93

Note: Mean ± SD values are computed within each question-type scoring scale (A/C: 0–1 point per item; K/A-II: 0–2 points per item) and should not be interpreted as directly comparable across formats without normalization. Cross-format comparisons are primarily based on normalized annual total score (Table S4) and type-specific accuracy (Table S5).

**Table 2:** Normality Test Results (Kolmogorov–Smirnov and Shapiro–Wilk) for LLM Scores Across Question Types.

Type	Model	Kolmogorov-Smirnova			Shapiro-Wilk		
		Statistic	df	Sig.	Statistic	df	Sig.
A-type	GPT_4o	0.504	1015	0.000	0.456	1015	0.000
	GPT_o1	0.533	1015	0.000	0.323	1015	0.000
	DeepSeek-V3	0.489	1015	0.000	0.495	1015	0.000
	DeepSeek_R1	0.494	1015	0.000	0.483	1015	0.000
C-type	GPT_4o	0.436	200	0.000	0.585	200	0.000
	GPT_o1	0.504	200	0.000	0.454	200	0.000
	DeepSeek-V3	0.428	200	0.000	0.593	200	0.000
	DeepSeek_R1	0.446	200	0.000	0.572	200	0.000
K-type	GPT_4o	0.369	280	0.000	0.632	280	0.000
	GPT_o1	0.502	280	0.000	0.46	280	0.000
	DeepSeek-V3	0.361	280	0.000	0.634	280	0.000
	DeepSeek_R1	0.441	280	0.000	0.578	280	0.000
A-II type	GPT_4o	0.439	220	0.000	0.581	220	0.000
	GPT_o1	0.479	220	0.000	0.518	220	0.000
	DeepSeek-V3	0.402	220	0.000	0.616	220	0.000
	DeepSeek_R1	0.434	220	0.000	0.586	220	0.000

Note: All *p*-values are significant at *p* < 0.001 (Lilliefors Significance Correction applied).

**Table 3:** Kruskal–Wallis Mean Ranks of Large Language Models by Question Type.

Type	Model	N	Mean Rank
A-type	GPT_4o	1015	2018
	GPT_o1	1015	2182
	DeepSeek-V3	1015	1950
	DeepSeek_R1	1015	1972
C-type	GPT_4o	200	385.5
	GPT_o1	200	443.5
	DeepSeek-V3	200	379.5
	DeepSeek_R1	200	393.5
K-type	GPT_4o	280	504.5
	GPT_o1	280	656.5
	DeepSeek-V3	280	496.5
	DeepSeek_R1	280	584.5
A-II	GPT_4o	220	440.5
	GPT_o1	220	476.5
	DeepSeek-V3	220	408.5
	DeepSeek_R1	220	436.5

Note: Mean Rank represents the average rank of each model in the Kruskal–Wallis test.

**Table 4:** Kruskal–Wallis H Test Results Comparing Model Performance Across Question Types.

Type	Kruskal–Wallis H	df	Asymp. Sig. (*p*-Value)
A-type	58.923	3	0
C-type	15.966	3	0.001
K-type	67.073	3	0
A-II type	12.416	3	0.006

Note: a. Kruskal–Wallis test was used to compare the distributions of different models (GPT-4o, GPT-o1, DeepSeek-V3, and DeepSeek-R1) within each type (A-type, C-type, K-type, A-II type). b. Grouping Variable: Model. c. A significant Asymp. Sig. (*p* < 0.05) indicates that at least one model differs.

**Table 5:** Pairwise Comparisons of Model Performance Across Question Types Using Mann–Whitney U Test with Bonferroni Correction.

Comparison	Type	Original *p*-Value	Bonferroni Corrected *p*-Value	Significant (α’ = 0.0083)
GPT-4o vs. GPT-o1	A-type	0.000	0.000 *	Yes
	C-type	0.001	0.006 *	Yes
	K-type	0.000	0.000 *	Yes
	A-II type	0.053	0.318	No
GPT-4o vs. DeepSeek-V3	A-type	0.000	0.000 *	Yes
	C-type	0.001	0.006 *	Yes
	K-type	0.000	0.000 *	Yes
	A-II type	0.053	0.318	No
GPT-4o vs. DeepSeek-R1	A-type	0.187	1.000	No
	C-type	0.664	1.000	No
	K-type	0.000	0.000 *	Yes
	A-II type	0.837	1.000	No
GPT-o1 vs. DeepSeek-V3	A-type	0.187	1.000	No
	C-type	0.664	1.000	No
	K-type	0.000	0.000 *	Yes
	A-II type	0.837	1.000	No
GPT-o1 vs. DeepSeek-R1	A-type	0.000	0.000 *	Yes
	C-type	0.003	0.018	No (0.018 > 0.0083)
	K-type	0.000	0.000 *	Yes
	A-II type	0.032	0.192	No
DeepSeek-V3 vs. DeepSeek-R1	A-type	0.541	1.000	No
	C-type	0.451	1.000	No
	K-type	0.000	0.000	Yes
	A-II type	0.162	0.972	No

Note: 1. Pairwise comparisons were conducted using the Mann–Whitney U test across four model comparisons (GPT-4o, GPT-o1, DeepSeek-V3, DeepSeek-R1) within each data type (A-type, C-type, K-type, and A-II type). 2. Bonferroni correction was applied to adjust for multiple comparisons (α′ = 0.05/6 ≈ 0.0083). 3. *p*-values are reported to three decimal places. Values reported as 0.000 indicate *p* < 0.001. 4. Only comparisons with Bonferroni corrected *p*-values less than 0.0083 are considered statistically significant. * denoted as *p* < 0.001

**Table 6:** Nonparametric Effect Sizes (r) for Pairwise Model Comparisons Across Question Types.

Comparison	Type	Z	N_total	r	Interpretation
GPT-4o vs. GPT-o1	A-type	−5.392	2030	0.12	Small effect
	C-type	−3.379	400	0.169	Small to moderate effect
	K-type	−6.933	560	0.293	Moderate effect
	A-II type	−1.935	440	0.092	Very small effect
GPT-4o vs. DeepSeek-V3	A-type	−5.392	2030	0.12	Small effect
	C-type	−3.379	400	0.169	Small to moderate effect
	K-type	−6.933	560	0.293	Moderate effect
	A-II type	−1.935	440	0.092	Very small effect
GPT-4o vs. DeepSeek-R1	A-type	−1.321	2030	0.029	Very small effect
	C-type	−0.434	400	0.022	Very small effect
	K-type	−3.488	560	0.147	Small effect
	A-II type	−0.205	440	0.01	Negligible effect
GPT-o1 vs. DeepSeek-V3	A-type	−1.321	2030	0.029	Very small effect
	C-type	−0.434	400	0.022	Very small effect
	K-type	−3.488	560	0.147	Small effect
	A-II type	−0.205	440	0.01	Negligible effect
GPT-o1 vs. DeepSeek-R1	A-type	−6.668	2030	0.148	Small to moderate effect
	C-type	−2.955	400	0.148	Small to moderate effect
	K-type	−3.562	560	0.151	Small effect
	A-II type	−2.138	440	0.102	Small effect
DeepSeek-V3 vs. DeepSeek-R1	A-type	−0.611	2030	0.014	Negligible effect
	C-type	−0.754	400	0.038	Very small effect
	K-type	−3.823	560	0.162	Small to moderate effect
	A-II type	−1.398	440	0.067	Very small effect

Note: 1. Effect size r is calculated as: $r=\left|Z\right|/\surd N(total)$. 2. Where N(total) is the total sample size for the two groups combined. 3. An r value of approximately 0.10 indicates a small effect, around 0.30 a moderate effect, and 0.50 or higher a large effect. 4. All values are rounded to three decimal places. These effect sizes supplement the *p* values by providing a measure of the practical significance of the differences between groups. Accordingly, statistically significant *p* values should be interpreted alongside effect sizes, as many significant contrasts may still reflect modest absolute accuracy differences with limited real-world impact.

## Data Availability

The examination questions used in this study are accessible via the official website. However, the authors do not own the examination content and are not authorized to grant permission for reuse or redistribution; therefore, the full question texts/images cannot be released or redistributed by the authors as a research dataset. To support reproducibility without redistributing protected exam content, derived item-level outcomes are provided as Supplementary Data S1 (Supplementary Data S1.xlsx) with an accompanying README (Supplementary Data S1_README.txt). Analysis code/scoring scripts are available from the corresponding author upon reasonable request. please contact dr.kaochiahung@gmail.com; 010040@tool.caaumed.org.tw.

## Supplementary Materials

The following supporting information can be downloaded at: https://www.mdpi.com/article/10.3390/bioengineering13030302/s1. Table S1. Taiwan Neurology Board Examination Composition by Year (Question Types and Total Items). Table S2. Changes in Score Contribution by Question Type in the Taiwan Neurology Board Examination (2018–2024). Table S3. Number of Correct Answers by Large Language Models (LLMs) Across Question Types in the Taiwan Neurology Board Examination (2018–2024). Table S4. Year-by-Year Performance of Large Language Models on the Taiwan Neurology Board Examination Format (2018–2024). Table S5. Accuracy Rates (%) of Large Language Models by Question Type in the Taiwan Neurology Board Examination (2018–2024). Table S6. Average Accuracy and Relative Difficulty Ranking of Question Types for Large Language Models (LLMs). Table S7. Comparison of Overall Average Accuracy by Question Type in Large Language Models: 2018–2023 vs. 2024. Table S8. Distribution of Exam Questions Containing Restricted Keywords and Their Impact on Score Loss by Year (2018–2024). Table S9. Pairwise Comparisons of Large Language Model Performance Using the Mann–Whitney U Test Across Question Types (A-type, C-type, K-type, and A-II-type) from 2018–2024 Taiwan Neurology Board Exams. Table S10. List of Board Examination Questions Rejected by DeepSeek-R1 Due to the Inclusion of the Keyword “Taiwan” (2018–2024). Figure S1. Full-stem screenshot used for DeepSeek input (question text + options + figure). Figure S2. DeepSeek response generated from a full-stem screenshot input. Figure S3. Image-only upload illustrating text-extraction failure in the DeepSeek interface. Figure S4. Example of keyword-triggered refusal in DeepSeek-R1 during evaluation (prompt containing “Taiwan”). Figure S5. Summary of model accuracy by question type (2018–2024 average). Figure S6. Evaluation workflow and platform-specific operational constraints.
