# Supplementary material for: Benchmarking Large Language Models on the Taiwan Neurology Board Examinations (2018–2024): A Comparative Evaluation of GPT-4o, GPT-o1, DeepSeek-V3, and DeepSeek-R1
Source: Bioengineering (Basel). 2026 Mar 5;13(3):302. doi: 10.3390/bioengineering13030302 (PMC13024452; doi:10.3390/bioengineering13030302)

Table S1. Taiwan Neurology Board Examination Composition by Year (Question Types and Total Items).

Year (CE)	Part I: A type	Part I: C type	Part II: K type	All type (image/table-only)	B type (case set)	Total items
2014	120	30	40	24	159	214
2015	120	30	40	25	120	215
2016	120	30	40	25	60	215
2017	135	30	40	25	60	230
2018	145	30	40	30	0	245
2019	145	30	40	30	0	245
2020	145	30	40	30	0	245
2021	145	30	40	30	0	245
2022	145	30	40	30	0	245
2023	145	30	40	30	0	245

Table note:

From 2018 onward, the annual item count and question-type composition were stable (total = 245; fixed A/C/K/All; B-type case sets absent), supporting longitudinal comparability. Pre-2018 examinations differed substantially in total items and included variable-sized B-type case sets.

Table S2. Changes in Score Contribution by Question Type in the Taiwan Neurology Board Examination (2018–2024).

Question Type	2018–2023 Contribution (%)	2024 Contribution (%)	Points per Question
A-type	46.03%	44.62%	1
C-type	9.52%	6.15%	1
K-type	25.40%	24.62%	2
A-II-type	19.05%	24.62%	2
Total	100% (315 points)	100% (325 points)	-

Table S3. Number of Correct Answers by Large Language Models (LLMs) Across Question Types in the Taiwan Neurology Board Examination (2018–2024).

Question Type	LLM Model	2018	2019	2020	2021	2022	2023	2024
A-type	GPT-4o	115	123	125	114	124	122	118
	GPT-o1	132	137	134	136	137	123	124
	DeepSeek-V3	103	119	121	117	119	120	108
	DeepSeek-R1	110	114	119	123	122	122	108
C-type	GPT-4o	22	23	19	20	21	18	14
	GPT-o1	26	27	23	22	27	26	15
	DeepSeek-V3	20	23	22	19	19	17	14
	DeepSeek-R1	23	24	21	21	19	19	14
K-type	GPT-4o	27	20	24	16	16	28	24
	GPT-o1	38	33	36	27	29	35	33
	DeepSeek-V3	24	20	20	18	16	31	22
	DeepSeek-R1	34	26	29	22	25	33	26
A-II-type	GPT-4o	21	25	20	22	19	20	25
	GPT-o1	24	26	22	22	25	20	31
	DeepSeek-V3	18	21	17	18	19	19	24
	DeepSeek-R1	20	19	20	20	24	22	24

Table S4. Year-by-Year Performance of Large Language Models on the Taiwan Neurology Board Examination Format (2018–2024).

Question Type	Model	2018	2019	2020	2021	2022	2023	2024
A-type (Single-choice, 1 point each)	GPT-4o	115.0	123.0	125.0	114.0	124.0	122.0	118.0
	GPT-o1	132.0	137.0	134.0	136.0	137.0	123.0	124.0
	DeepSeek-V3	103.0	119.0	121.0	117.0	119.0	120.0	108.0
	DeepSeek-R1	110.0	114.0	119.0	123.0	122.0	122.0	108.0
C-type (Multiple-choice, 1 point each)	GPT-4o	22.0	23.0	19.0	20.0	21.0	18.0	14.0
	GPT-o1	26.0	27.0	23.0	22.0	27.0	26.0	15.0
	DeepSeek-V3	20.0	23.0	22.0	19.0	19.0	17.0	14.0
	DeepSeek-R1	23.0	24.0	21.0	21.0	19.0	19.0	14.0
K-type (True-false multiple-choice, 2 points each)	GPT-4o	42.0	50.0	40.0	44.0	38.0	40.0	50.0
	GPT-o1	48.0	52.0	44.0	44.0	50.0	40.0	62.0
	DeepSeek-V3	36.0	42.0	34.0	36.0	38.0	38.0	48.0
	DeepSeek-R1	40.0	38.0	40.0	40.0	48.0	44.0	48.0
A-II-type (Image-based single-choice, 2 points each)	GPT-4o	54.0	40.0	48.0	32.0	32.0	56.0	48.0
	GPT-o1	76.0	66.0	72.0	54.0	58.0	70.0	66.0
	DeepSeek-V3	48.0	40.0	40.0	36.0	32.0	62.0	44.0
	DeepSeek-R1	68.0	52.0	58.0	44.0	50.0	66.0	52.0
Overall Score (out of 100)	GPT-4o	74.0	74.9	73.7	66.7	68.3	74.9	70.8
	GPT-o1	89.5	89.5	86.7	81.3	86.3	82.2	82.2
	DeepSeek-V3	65.7	71.1	68.9	66.0	66.0	75.2	65.8
	DeepSeek-R1	76.5	72.4	75.6	72.4	75.9	79.7	68.3

Notes:

1. Question Structure:
   - A-type: Single-choice questions (145 questions per year, 1 point each).
   - C-type: Multiple-choice questions (30 questions per year from 2018 to 2023, reduced to 20 questions in 2024, 1 point each).
   - K-type: True-false multiple-choice questions (40 questions per year, 2 points each).
   - A-II-type: Image-based single-choice questions (30 questions per year from 2018 to 2023, increased to 40 questions in 2024, 2 points each).
2. Scoring System:

   Total maximum points: 315 from 2018 to 2023, 325 in 2024.

   Final scores are calculated as: (Raw Score / Total Maximum Points) × 100.

Table S5. Accuracy Rates (%) of Large Language Models by Question Type in the Taiwan Neurology Board Examination (2018–2024).

Question Type	LLM Model	2018	2019	2020	2021	2022	2023	2024	Average
A-type	GPT-4o	79.31%	84.83%	86.21%	78.62%	85.52%	84.14%	81.38%	82.86%
	GPT-o1	91.03%	94.48%	92.41%	93.79%	94.48%	84.83%	85.52%	91.84%
	DeepSeek-V3	71.03%	82.07%	83.45%	80.69%	82.07%	82.76%	74.48%	79.51%
	DeepSeek-R1	75.86%	78.62%	82.07%	84.83%	84.14%	84.14%	74.48%	80.59%
C-type	GPT-4o	73.33%	76.67%	63.33%	66.67%	70.00%	60.00%	70.00%	68.57%
	GPT-o1	86.67%	90.00%	76.67%	73.33%	90.00%	86.67%	75.00%	82.62%
	DeepSeek-V3	66.67%	76.67%	73.33%	63.33%	63.33%	56.67%	70.00%	67.14%
	DeepSeek-R1	76.67%	80.00%	70.00%	70.00%	63.33%	63.33%	70.00%	70.48%
K-type	GPT-4o	67.50%	50.00%	60.00%	40.00%	40.00%	70.00%	60.00%	55.36%
	GPT-o1	95.00%	82.50%	90.00%	67.50%	72.50%	87.50%	82.50%	82.50%
	DeepSeek-V3	60.00%	50.00%	50.00%	45.00%	40.00%	77.50%	55.00%	53.93%
	DeepSeek-R1	85.00%	65.00%	72.50%	55.00%	62.50%	82.50%	65.00%	69.64%
A-II-type	GPT-4o	70.00%	83.33%	66.67%	73.33%	63.33%	66.67%	62.50%	69.40%
	GPT-o1	80.00%	86.67%	73.33%	73.33%	83.33%	66.67%	77.50%	77.26%
	DeepSeek-V3	60.00%	70.00%	56.67%	60.00%	63.33%	63.33%	60.00%	61.90%
	DeepSeek-R1	66.67%	63.33%	66.67%	66.67%	80.00%	73.33%	60.00%	68.10%
Average	GPT-4o	72.54%	73.71%	69.05%	64.66%	64.71%	70.20%	68.47%	69.05%
	GPT-o1	88.18%	88.41%	83.10%	76.99%	85.08%	81.42%	80.13%	83.86%
	DeepSeek-V3	64.43%	69.68%	65.86%	62.26%	62.18%	70.06%	64.87%	65.62%
	DeepSeek-R1	76.05%	71.74%	72.81%	69.12%	72.49%	75.83%	67.37%	72.20%

Notes:
This table shows the percentage of correctly answered questions for each question type and model Percentages represent the ratio of correct answers to the total number of questions in each category The "Average" row shows the overall performance across all question types for each model

Table S6. Average Accuracy and Relative Difficulty Ranking of Question Types for Large Language Models (LLMs).

Question Type (Avg.)	GPT-4o	GPT-o1	DeepSeek-V3	DeepSeek-R1	Overall Avg.	Difficulty Rank
A-type	82.86%	91.84%	79.51%	80.59%	83.70%	4 (Easiest)
C-type	68.57%	83.89%	67.14%	70.48%	72.52%	3
K-type	55.36%	82.50%	53.93%	69.64%	65.36%	1 (Hardest)
A-II-type	69.40%	77.22%	61.90%	68.10%	69.16%	2

Table S7. Comparison of Overall Average Accuracy by Question Type in Large Language Models: 2018–2023 vs. 2024.

Question Type	2018–2023 Avg.	2024 Avg.
A-type	84.23%	76.78%
C-type	72.36%	70.00%
K-type	65.31%	60.00%
A-II-type	69.86%	60.83%

2018–2023: A-type is the easiest (84.23%), K-type the hardest (65.31%).

2024: A-type remains the easiest (76.78%), K-type the hardest (60.00%).

The previous difficulty ranking (K-type > A-II-type > C-type > A-type) still holds based on the accuracy data

Table S8. Distribution of Exam Questions Containing Restricted Keywords and Their Impact on Score Loss by Year (2018–2024).

Part A. Restricted-keyword questions and score impact.

Question Type	2018	2019	2020	2021	2022	2023	2024
A-type	2	5	2	5	4	5	7
C-type	0	1	0	0	0	0	1
K-type	0	2	1	2	0	0	0
A-II-type	3	1	0	1	0	1	1
Total Questions with Restrictions	5	9	3	8	4	6	9
Total Score Lost	8	12	4	11	4	7	10
Percentage of Total Points Lost	2.54%	3.81%	1.27%	3.49%	1.27%	2.22%	3.08%

Part B. Sensitivity analysis (DeepSeek-R1; excluding refusals).

Metric	2018	2019	2020	2021	2022	2023	2024
Observed raw score (points)	241	228	238	228	239	251	222
Original normalized score (%) (Table S4)	76.5	72.4	75.6	72.4	75.9	79.7	68.3
Normalized score excluding refused items (%)	81.1	79.2	77.8	78.6	78.1	83.8	73.7

Table note: As a sensitivity analysis, we recalculated DeepSeek-R1 performance after excluding refusals from the denominator to reflect performance on answerable items, alongside the original standardized scoring. The refusal-excluded normalized score was computed as: Normalized score excluding refusals (%) = ((S + L) / (M - L)) × 100, where S is the observed raw score, L is the total score lost due to refusals (Part A), and M is the annual maximum points (315 for 2018–2023; 325 for 2024).

Table S9. Pairwise Comparisons of Large Language Model Performance Using the Mann–Whitney U Test Across Question Types (A-type, C-type, K-type, and A-II-type) from 2018–2024 Taiwan Neurology Board Exams.

1. GPT-4o vs GPT-o1

**Ranks**					
Type		Model_NO	N	Mean Rank	Sum of Ranks
A-II type	Value	GPT-4o	220	211.50	46530.00
		GPT-o1	220	229.50	50490.00
		Total	440		
A-type	Value	GPT-4o	1015	974.50	989117.50
		GPT-o1	1015	1056.50	1072347.50
		Total	2030		
C-type	Value	GPT-4o	200	186.00	37200.00
		GPT-o1	200	215.00	43000.00
		Total	400		
K-type	Value	GPT-4o	280	242.50	67900.00
		GPT-o1	280	318.50	89180.00
		Total	560		

**Test Statistics[a]**		
Type		Value
A-II type	Mann-Whitney U	22220.000
	Wilcoxon W	46530.000
	Z	-1.935
	Asymp. Sig. (2-tailed)	.053
A-type	Mann-Whitney U	473497.500
	Wilcoxon W	989117.500
	Z	-5.392
	Asymp. Sig. (2-tailed)	.000
C-type	Mann-Whitney U	17100.000
	Wilcoxon W	37200.000
	Z	-3.379
	Asymp. Sig. (2-tailed)	.001
K-type	Mann-Whitney U	28560.000
	Wilcoxon W	67900.000
	Z	-6.933
	Asymp. Sig. (2-tailed)	.000
a. Grouping Variable: Model_NO		

2. GPT-4o vs DeepSeek-V3

Ranks					
Type		Model_NO	N	Mean Rank	Sum of Ranks
A-II type	Value	GPT-4o	220	228.50	50270.00
		DeepSeek-V3	220	212.50	46750.00
		Total	440		
A-type	Value	GPT-4o	1015	1032.50	1047987.50
		DeepSeek-V3	1015	998.50	1013477.50
		Total	2030		
C-type	Value	GPT-4o	200	202.00	40400.00
		DeepSeek-V3	200	199.00	39800.00
		Total	400		
K-type	Value	GPT-4o	280	282.50	79100.00
		DeepSeek-V3	280	278.50	77980.00
		Total	560		

Test Statistics[a]		
Type		Value
A-II type	Mann-Whitney U	22220.000
	Wilcoxon W	46530.000
	Z	-1.935
	Asymp. Sig. (2-tailed)	0.053
A-type	Mann-Whitney U	473497.500
	Wilcoxon W	989117.500
	Z	-5.392
	Asymp. Sig. (2-tailed)	0.000
C-type	Mann-Whitney U	17100.000
	Wilcoxon W	37200.000
	Z	-3.379
	Asymp. Sig. (2-tailed)	0.001
K-type	Mann-Whitney U	28560.000
	Wilcoxon W	67900.000
	Z	-6.933
	Asymp. Sig. (2-tailed)	0.000
a. Grouping Variable: Model_NO		

3. GPT-4o vs DeepSeek-R1

Ranks					
Type		Model_NO	N	Mean Rank	Sum of Ranks
A-II type	Value	GPT-4o	220	221.50	48730.00
		DeepSeek-R1	220	219.50	48290.00
		Total	440		
A-type	Value	GPT-4o	1015	1027.00	1042405.00
		DeepSeek-R1	1015	1004.00	1019060.00
		Total	2030		
C-type	Value	GPT-4o	200	198.50	39700.00
		DeepSeek-R1	200	202.50	40500.00
		Total	400		
K-type	Value	GPT-4o	280	260.50	72940.00
		DeepSeek-R1	280	300.50	84140.00
		Total	560		

Test Statistics[a]		
Type		Value
A-II type	Mann-Whitney U	23980.000
	Wilcoxon W	48290.000
	Z	-.205
	Asymp. Sig. (2-tailed)	.837
A-type	Mann-Whitney U	503440.000
	Wilcoxon W	1019060.000
	Z	-1.321
	Asymp. Sig. (2-tailed)	.187
C-type	Mann-Whitney U	19600.000
	Wilcoxon W	39700.000
	Z	-.434
	Asymp. Sig. (2-tailed)	.664
K-type	Mann-Whitney U	33600.000
	Wilcoxon W	72940.000
	Z	-3.488
	Asymp. Sig. (2-tailed)	.000
a. Grouping Variable: Model_NO		

4. GPT-o1vs DeepSeek-V3

Ranks					
Type		Model_NO	N	Mean Rank	Sum of Ranks
A-II type	Value	GPT-o1	220	237.50	52250.00
		DeepSeek-V3	220	203.50	44770.00
		Total	440		
A-type	Value	GPT-o1	1015	1073.50	1089602.50
		DeepSeek-V3	1015	957.50	971862.50
		Total	2030		
C-type	Value	GPT-o1	200	216.50	43300.00
		DeepSeek-V3	200	184.50	36900.00
		Total	400		
K-type	Value	GPT-o1	280	320.50	89740.00
		DeepSeek-V3	280	240.50	67340.00
		Total	560		

Test Statistics[a]		
Type		Value
A-II type	Mann-Whitney U	23980.000
	Wilcoxon W	48290.000
	Z	-.205
	Asymp. Sig. (2-tailed)	.837
A-type	Mann-Whitney U	503440.000
	Wilcoxon W	1019060.000
	Z	-1.321
	Asymp. Sig. (2-tailed)	.187
C-type	Mann-Whitney U	19600.000
	Wilcoxon W	39700.000
	Z	-.434
	Asymp. Sig. (2-tailed)	.664
K-type	Mann-Whitney U	33600.000
	Wilcoxon W	72940.000
	Z	-3.488
	Asymp. Sig. (2-tailed)	.000
a. Grouping Variable: Model_NO		

5. GPT-o1vs DeepSeek-R1

Ranks					
Type		Model_NO	N	Mean Rank	Sum of Ranks
A-II type	Value	GPT-o1	220	230.50	50710.00
		DeepSeek-R1	220	210.50	46310.00
		Total	440		
A-type	Value	GPT-o1	1015	1068.00	1084020.00
		DeepSeek-R1	1015	963.00	977445.00
		Total	2030		
C-type	Value	GPT-o1	200	213.00	42600.00
		DeepSeek-R1	200	188.00	37600.00
		Total	400		
K-type	Value	GPT-o1	280	298.50	83580.00
		DeepSeek-R1	280	262.50	73500.00
		Total	560		

Test Statistics[a]		
Type		Value
A-II type	Mann-Whitney U	22000.000
	Wilcoxon W	46310.000
	Z	-2.138
	Asymp. Sig. (2-tailed)	.032
A-type	Mann-Whitney U	461825.000
	Wilcoxon W	977445.000
	Z	-6.668
	Asymp. Sig. (2-tailed)	.000
C-type	Mann-Whitney U	17500.000
	Wilcoxon W	37600.000
	Z	-2.955
	Asymp. Sig. (2-tailed)	.003
K-type	Mann-Whitney U	34160.000
	Wilcoxon W	73500.000
	Z	-3.562
	Asymp. Sig. (2-tailed)	.000
a. Grouping Variable: Model_NO		

6. DeepSeek-V3 vs DeepSeek-R1

Ranks					
Type		Model_NO	N	Mean Rank	Sum of Ranks
A-II type	Value	DeepSeek-V3	220	213.50	46970.00
		DeepSeek-R1	220	227.50	50050.00
		Total	440		
A-type	Value	DeepSeek-V3	1015	1010.00	1025150.00
		DeepSeek-R1	1015	1021.00	1036315.00
		Total	2030		
C-type	Value	DeepSeek-V3	200	197.00	39400.00
		DeepSeek-R1	200	204.00	40800.00
		Total	400		
K-type	Value	DeepSeek-V3	280	258.50	72380.00
		DeepSeek-R1	280	302.50	84700.00
		Total	560		

Test Statistics[a]		
Type		Value
A-II type	Mann-Whitney U	22660.000
	Wilcoxon W	46970.000
	Z	-1.398
	Asymp. Sig. (2-tailed)	.162
A-type	Mann-Whitney U	509530.000
	Wilcoxon W	1025150.000
	Z	-.611
	Asymp. Sig. (2-tailed)	.541
C-type	Mann-Whitney U	19300.000
	Wilcoxon W	39400.000
	Z	-.754
	Asymp. Sig. (2-tailed)	.451
K-type	Mann-Whitney U	33040.000
	Wilcoxon W	72380.000
	Z	-3.823
	Asymp. Sig. (2-tailed)	.000
a. Grouping Variable: Model_NO		

Table S10. List of Board Examination Questions Rejected by DeepSeek-R1 Due to the Inclusion of the Keyword 'Taiwan' (2018–2024).

Type	Year	Question No	Question with Options
A-type	2018	33	Which of the following descriptions is FALSE?<br>A. PARK4 is related to duplication or triplication at the synuclein alpha gene (SNCA).<br>B. The clinical features of SNCA mutation include dementia and autonomic dysfunction.<br>C. G2385R is the most common LRRK2 mutation in Taiwan.<br>D. No Lewy body inclusions are found in most parkinsonism patients with GBA mutations.<br>E. GCH1 mutation is a risk factor of Parkinson disease.
A-type	2018	81	Which one of the following statements about familial amyloidosis polyneuropathy in Taiwan is FALSE?<br>A. the most comment mutation site is TTR Ala97Ser mutation.<br>B. complete penetration in the family<br>C. cardiac involvement is frequently seen after age of 55 years<br>D. usually associated with carpal tunnel syndrome<br>E. Autonomic dysfunction is commonly seen.
A-type	2019	31	According to 2019 Taiwan Stroke Society Guideline, intravenous thrombolysis is NOT suitable in which one of the following conditions?<br>A. Aged 79, 4 hours after onset, NIHSS 25, without other contraindications<br>B. Aged 81, 4 hours after onset, NIHSS 13, without other contraindications<br>C. Aged 70, 4 hours after onset, NIHSS 18, INR 1.8, without other contraindications<br>D. Aged 65, 2 hours after onset, NIHSS 22 improved to 17, without other contraindications<br>E. Aged 85, 2 hours after onset, NIHSS 18, INR 1.5, without other contraindications
A-type	2019	69	A 28-year-old female patient with temporal lobe epilepsy presented with skin rash over trunk after starting lamotrigine therapy for one week. Which one of the following statements is FALSE?<br>A. Antiepileptic drugs (AED) with aromatic rings structure e.g. carbamazepine and phenytoin should be avoided in this patient.<br>B. HLA-B*31:01 is associated with lamotrigine-induced Stevens–Johnson syndrome/Toxic epidermal necrolysis (SJS/TEN) in Taiwan.<br>C. HLA-B*15:02 is associated with carbamazepine-induced SJS/TEN in Taiwan.<br>D. Lacosamide may carry a low risk of AED-

			induced cutaneous adverse effects.<br>E. Oxcarbazepine-SJS has less clinical severity and better clinical outcomes than carbamazepine-SJS dose.
A-type	2019	96	Which one of the statements about spinocerebellar ataxia (SCA) is TRUE?<br>A. SCA5 is due to trinucleotide repeat expansion.<br>B. Anticipation phenomenon is frequently seen in SCA6.<br>C. Epilepsy is frequently seen in patients with late onset Dentatorubral-Pallidoluysian Atrophy<br>D. Retinal photoreceptor degeneration is frequently seen in SCA7<br>E. SCA6 is the most common SCA subtype in Taiwan.
A-type	2019	136	Which one of the statements about “CADASIL” is TRUE?<br>A. CADASIL is caused by a mutation in the NOTCH1 gene.<br>B. Patients with CADASIL may have cerebral microbleeds.<br>C. The clinical manifestations of CADASIL include lower limbs weakness with fasciculation.<br>D. All the patients with CADASIL have leukoencephalopathy involving anterior temporal regions.<br>E. Most patients with CADASIL in Taiwan have ischemic strokes before age 40 years.
A-type	2019	137	Which one of the following statements is FALSE?<br>A. The most common subtype of Charcot-Marie-Tooth disease is CMT1A.<br>B. The most common subtype of hereditary spastic paraplegia is SPG1.<br>C. The most common subtype of familial amyloidotic neuropathy is caused by a mutation in the TTR gene.<br>D. The most common subtype of spinocerebellar ataxia in in Taiwan is SCA3.<br>E. The most common subtype of limb-girdle muscular dystrophy is LGMD2A
A-type	2020	136	Hereditary transthyretin amyloidosis with polyneuropathy, also known as family amyloid polyneuropathy (FAP), is characterized by extracellular deposition of amyloid and destruction of the somatic and autonomic peripheral nervous system. Which of the following statements about FAP is FALSE?<br>A. The most common reported mutation in Taiwan is Ala97Ser mutation<br>B. With cardiac involvement, sparkling appearance in myocardium might be seen on echocardiography

			C. Patisiran, an RNAi drug, showed promising benefits in improving clinical manifestations<br>D. The negative results of salivary gland biopsy or abdominal fat biopsy does not eliminate the diagnosis of FAP<br>E. The deterioration of cardiomyopathy usually stops after liver transplantation
A-type	2020	139	Which of the following statements about Charcot-Marie-Tooth disease is FALSE?<br>A. Present as progressive distal weakness and atrophy, foot deformities, distal sensory loss, and depressed tendon reflexes<br>B. The most common axonal type CMT (CMT2) in Taiwan is caused by NEFL mutation<br>C. CMT type 1 is a group of autosomal dominant-inherited demyelinating neuropathies<br>D. In adult, the most common form of CMT in Taiwan is associated with PMP 22 duplication on chromosome 17p11.2<br>E. Currently there is no cure or effective disease-modifying treatment
A-type	2021	10	According to the payment rule of Taiwan National Health insurance, which oral migraine preventive medicine is needed to be attempted before application for NHI-paid botulinum toxin or anti-CGRP treatment?<br>A. Valproic acid<br>B. Lithium<br>C. Amitriptyline<br>D. Propranolol<br>E. Topiramate
A-type	2021	40	Which of the following is FALSE regarding to 2020 Taiwan Stroke Society guidelines for BP control in ischemic stroke?<br>A. For patients receiving endovascular thrombectomy, if the mean arterial BP values cannot be obtained during the procedure, it is reasonable to control the systolic BP between 140-180mmHg (COR: IIa, LOE: C-EO).<br>B. For patients receiving endovascular thrombectomy, the BP target within 24 hours after procedure is depending on the status of reperfusion.<br>C. After the acute phase of ischemic stroke caused by large artery atherosclerosis, it is recommended to start treatment with a target BP <160/90mmHg (COR:I, LOE:B-R).<br>D. For primary prevention of stroke in hypertensive patients over 75 years of age, it is reasonable to control the target BP below 140/90 mmHg (COR:IIa, LOE:B-R).
A-type	2021	107	Which of the following statements about amyotrophic lateral sclerosis (ALS) genetics is correct?

			A. More than 50% of ALS patients have a familial history of ALS.<br>B. The GGGGCC repeat expansion in C9ORF72 gene is the most common cause of familial ALS globally.<br>C. All the familial ALS are dominantly inherited.<br>D. Mutations in FUS gene are the most common cause of familial ALS in Taiwan.<br>E. ALS patients with a SOD1 mutation usually also have frontotemporal dementia.
A-type	2021	109	Which of the following statements about inherited cerebral small vessel disease is incorrect?<br>A. Mutations in HTRA1 gene may cause both autosomal dominant and autosomal recessive cerebral small vessel diseases.<br>B. NOTCH3 mutations are associated cerebral microbleeds but not intracerebral hemorrhage.<br>C. Alopecia and spinal spondylosis are common clinical features in patients with cerebral autosomal recessive arteriopathy with subcortical infarcts and leukoencephalopathy (CARASIL).<br>D. Most CADASIL patients have a cysteine-altering NOTCH3 mutation.<br>E. In Taiwan, approximately 50% of CADASIL patients have T2-weighted MRI hyperintensities involving anterior temporal regions.
A-type	2021	123	What is WRONG about the statement of familial amyloidosis polyneuropathy in Taiwan<br>A, the most comment mutation site is Ala97Ser<br>B, complete penetration in family<br>C, cardiac involvement is usual after age of 55 years<br>D, no curable treatment is available at present<br>E, autonomic dysfunction is common
A-type	2022	54	Which of the following is NOT correct regarding to 2020 Taiwan Stroke Society guidelines for BP control in ischemic stroke?<br>A. For patients receiving endovascular thrombectomy, if the mean arterial BP values cannot be obtained during the procedure, it is reasonable to control the systolic BP between 140-180mmHg (COR: IIa, LOE: C-EO).<br>B. For patients receiving endovascular thrombectomy, the BP target within 24 hours after procedure is depending on the status of reperfusion.<br>C. After the acute phase of ischemic stroke caused by large artery atherosclerosis, it is recommended to start treatment with a target BP <160/90mmHg (COR:I, LOE:B-R).<br>D. For primary prevention of stroke in

			hypertensive patients over 75 years of age, it is reasonable to control the target BP below 140/90 mmHg (COR:IIa, LOE:B-R).
A-type	2022	61	Which of the following statement is FALSE about CADASIL patients in Taiwan?<br>A. Most common genetic mutation is NOTCH3 p.R544C<br>B. Most of the patients initially present with acute ischemic stroke or transient ischemic attack<br>C. Most common site for white matter hyperintensities on brain MRI is the anterior temporal lobe<br>D. Cognition impairment is not uncommon among patients<br>E. Microbleeds are common findings on brain MRI
A-type	2022	110	Which of the following statement is FALSE about cerebellar ataxia?<br>A. SCA3 is the most common subtype of spinocerebellar ataxia in Taiwan, followed by SCA2.<br>B. Some of the SCA2 patients may manifest with parkinsonism feature at the early stage of disease.<br>C. SCA17 patients usually had a cognitive decline and psychiatric symptoms<br>D. Fragile X tremor-ataxic syndrome is characterized by progressive gait ataxia since childhood or adolescence.<br>E. All of the above are correct
A-type	2022	138	Which of the following statement is FALSE?<br>A. The split hand syndrome can be also found in amyotrophic lateral sclerosis (ALS).<br>B. Tomacula is characteristic of sural nerve biopsy of hereditary neuropathy with pressure palsies (HNPP)<br>C. PMP22 duplication may cause hereditary neuropathy with pressure palsies (HNPP)<br>D. Uniform conduction slowing is often used to distinguish CMT1 from acquired demyelinating neuropathies<br>E. PMP22 and GJB1 mutations are commonly seen in Taiwanese patients with CMT1
A-type	2023	44	Which statement about cannabidiol (CBD) is FALSE?<br>A. It is an active ingredient in cannabis (marijuana)<br>B. Taiwan approved CBD for treating Dravet syndrome and Lennox-Gastaunt syndrome<br>C. CBD can increase the concentration of clobazam<br>D. It has been approved to be effective for seizure control in patients with tuberous sclerosis

			E. CBD is psychoactive drug from the cannabis plant
A-type	2023	70	Which of the following is FALSE for Guillain-Barre syndrome (GBS)?<br>A. The Miller-Fisher syndrome (MFS) variant accounts for 6% of total GBS cases in Taiwan, whereas in Western countries the proportion is as high as 18%.<br>B. Increased protein in CSF without pleocytosis (albuminocytological dissociation).<br>C. The maximal deficits develop over days or weeks (maximum 4 weeks), followed by a plateau phase and gradual recovery.<br>D. Plasma exchange and high-dose IVIG infusions have been shown to be equally effective.<br>E. The most common identifiable bacterial organism linked to GBS and particularly its axonal forms is C. jejuni.
A-type	2023	80	Which is FALSE about the statement of familial amyloidosis polyneuropathy in Taiwan?<br>A.the most common mutation site is Ala97Ser<br>B.complete penetration in family<br>C.cardiac involvement is usual after age of 55 years<br>D.no curable treatment is available at present<br>E.autonomic dysfunction is common
A-type	2023	93	For the preventive treatment of episodic migraine, which of the following belong to the category of first-line agents in the Taiwan Headache Society guidelines?<br>A. Verapamil<br>B. Valproic acid<br>C. Amitriptyline<br>D. Galcanezumab<br>E. Topiramate
A-type	2023	126	Which of the following statements about spinocerebellar ataxia (SCA) is FALSE?<br>A.Retinal degeneration is a characteristic feature of SCA type 17.<br>B.Machado-Joseph disease is the most common SCA subtype in Taiwan.<br>C. SCA type 10 is not one of the trinucleotide repeat expansion diseases.<br>D. Patients with SCA type 2 may also have neuropathy and ophthalmoparesis.<br>E. Mutations in the potassium channel genes, including KCNC3 and KCND3, may cause SCA
A-type	2023	99	What is the TRUE description for cluster headache and trigeminal autonomic cephalalgias?<br>A. Galcanezumab, administered with 300mg monthly subcutaneous injection, has been shown effective in randomized controlled trials for the

			preventive treatment of both episodic and chronic cluster headaches.<br>B. Inhaled oxygen at 12L/min via a nasal cannula could be used for acute treatment for cluster headache attacks.<br>C. According to the 2022 Taiwan Headache Society Guidelines for Acute and Preventive Treatment of Cluster Headaches, propranolol at a dosage of 40-160mg/day is recommended as a preventive treatment for cluster headaches.<br>D. According to the ICHD-3, the diagnosis of 'hemicrania continua, unremitting subtype', also requires 'absolutely 'responsiveness to therapeutic doses of indomethacin, typically required 150 mg/day.<br>E. In ICHD-3, the diagnosis of short-lasting unilateral neuralgiform headache attacks with cranial autonomic symptoms (SUNA) required the presence of both conjunctival injection and lacrimation ipsilateral to the pain side.
A-type	2024	3	A 43 years old male had suffered from acute brainstem stroke. In the young stroke survey, deficiency of α - galactosidase A activity was noted. Which of the following about his disease is FALSE?<br>A. The common MRI findings are white matter hyperintensity and basilar artery dolichoectasia.<br>B. Patients typically have painful neuropathy and skin angiokeratomas in childhood then cerebral, renal and heart disease in 30-50 years of age.<br>C. It is a hereditary disease of X-linked pattern. Female patients usually have more early and severe symptoms than male.<br>D. In Taiwan, most of the patients of this disease are cardiac variant, who usually present unexplained left ventricular hypertrophy and lack of other typical symptoms.<br>E. Enzyme replacement therapy may help to delay the vasculopathy development in cerebral vascular system.
A-type	2024	41	Which of the following statements regarding Dravet syndrome is FALSE?<br>A. 80% of the syndrome is caused by sodium channel gene SCN1A mutations.<br>B. Usually onset before age 1 with febrile convulsion or febrile status epilepticus.<br>C. Adult Dravet syndrome may present with parkinsonism-like gait (crouch gait).<br>D. Sodium channel blocker should be the first line treatment.<br>E. Cannabidiol and Stiripental are indicated for this syndrome in Taiwan.

A-type	2024	80	Which of the following is FALSE about S1P receptor modulators?<br>A. S1PR5 promote natural killer cell trafficking.<br>B. Fingolimod worked as S1PR2 antagonist to inhibit immune cell migration.<br>C. Sipolimod is the only approved DMT to use on patients with SPMS in Taiwan.<br>D. Patients under S1P receptor modulators should follow liver function test per 3 months<br>E. Patients under fingolimod may develop severe side effect of progressive multifocal leukoencephalopathy.
A-type	2024	100	According to the 2022 Taiwan Guidelines for Preventive Treatment of Migraine, which of the following statements is TRUE?<br>A. Using topiramate as a preventive medication for 6 months and then discontinuing it may lead to an increase in headache frequency, returning to pre-treatment levels within six months after stopping.<br>B. Lamotrigine is effective in preventing the frequency of headache attacks.<br>C. The effectiveness of botulinum toxin in preventing chronic migraine is currently thought to be related to its muscle-relaxing properties.<br>D. The impact of valproate and flunarizine on weight is the same.<br>E. For migraine prevention, the evidence for aerobic exercise is equivalent to that for resistance exercise.
A-type	2024	130	Which of the following statements about spinocerebellar ataxia (SCA) is FALSE?<br>A. Retinal degeneration is a characteristic feature of SCA type 7.<br>B. Machado-Joseph disease is the most common SCA subtype in Taiwan.<br>C. CAG repeat numbers of mutant SCA6 alleles are usually larger than those of mutant SCA1 alleles.<br>D. Patients with SCA type 3 may have parkinsonism and/or neuropathy in addition to cerebellar ataxia.<br>E. Pontine atrophy is common in patients with SCA2.
A-type	2024	131	Which of the following statement about familial spastic paraplegia is TRUE?<br>A. Spastic paraplegia type 1 (SPG1) is the most common subtype of hereditary spastic paraplegia (HSP)<br>B. Mutations in the ABCD1 gene may lead to autosomal dominant spastic paraplegia syndrome.<br>C. SPG5, cause by mutations in the CYP7B1 gene, is the most common autosomal dominant

			spastic paraplegia syndrome in Taiwan.<br>D. Point mutations in the SPAST gene are the only cause of SPG4.<br>E. In addition to spastic paraplegia, some patients with HSP may also manifest cognitive impairment.
C-type	2019	8	According to the amended 'Regulations for Governing the Management of Medical Device in Taiwan' in September 2018, which of the following cell type(s) is (are) the optional treatment for chronic ischemic stroke?<br>A. Autologous peripheral blood CD34+ stem cells<br>B. Autologous bone marrow-derived mesenchymal stem cells
C-type	2024	14	Regarding the migraine aura, which statement(s) is(are) TRUE?<br>A. According to the ICHD-3, the retinal migraine is a subtype of migraine with aura, with binocular visual disturbance, including scintillations, scotomata or blindness, associated with migraine headache.<br>B. According to 2022 Taiwan Guidelines for Preventive Treatment of Migraine, lamotrigine is effective in reducing headache frequency in migraine with aura
K-type	2019	3	According to the "medical treatment guidelines for preventive treatment of migraine" by Taiwan Headache Society in 2017, which one(s) of the following statements about migraine prevention is (are) TRUE?<br>1. Generally, medication for migraine prevention is not suggested in pregnant woman<br>2. Non-medication treatment is firstly recommended in elderly because elder patients may have taken multiple medications, and have high risks of adverse effects<br>3. Triptans or NSAIDs is firstly considered in short-term prevention of menstrual migraine<br>4. Oral contraceptive combined estrogen and progesterone is firstly considered in short-term prevention of menstrual migraine
K-type	2019	28	Which statement(s) about genetic parkinsonism is (are) TRUE?<br>1. Exonic deletions are common mutation types for Parkin gene<br>2. LRRK2 G2019S is a common genetic cause of autosomal dominant Parkinson's disease in Taiwan.<br>3. The mutation types for SNCA gene in autosomal dominant Parkinson's disease include point mutations and duplication.<br>4. Dystonia is a rare presentation in PARK2 patients

K-type	2020	10	Which of the following(s) is(are) TRUE about cerebral autosomal dominant arteriopathy with subcortical infarcts and leukoencephalopathy (CADASIL)?<br>1. p.R544C in exon 11 is the most common mutation in Taiwan CADASIL population<br>2. Patients with CADASIL would develop both lacunar infarction and intracerebral hemorrhage<br>3. In Taiwan CADASIL population, pathognomonic temporal leukoaraiosis is not as common as western CADASIL population<br>4. Large artery atherosclerosis is an exclusion criteria of CADASIL diagnosis
K-type	2021	13	Which of the following(s) are true about cerebral autosomal dominant arteriopathy with subcortical infarcts and leukoencephalopathy (CADASIL)?<br>1 p.R544C in exon 11 is the most common mutation in Taiwan CADASIL population<br>2 Patients with CADASIL would develop both lacunar infarction and intracerebral hemorrhage<br>3 In Taiwan CADASIL population, pathognomonic temporal leukoaraiosis is not as common as in western CADASIL population<br>4 Large artery atherosclerosis is an exclusion criteria of CADASIL diagnosis
K-type	2021	37	Which description about familial amyloid neuropathy (FAP) is TRUE ?<br>1. Small fiber neuropathy frequently is the initial presentation<br>2. Monoclonal gammopathy is usually associated<br>3. Val30Met is the most common transthyretin mutation (TTR-FAP) globally<br>4. Late onset (more than 50 year-old) TTR-FAP usually have negative family history in Taiwan
A-II	2018	14	A 12 year-old Taiwanese girl had scoliosis and wasting of distal limbs since her childhood. No family history can be traced. Her illness was not responsive to steroid therapy. Her nerve conduction study and sural nerve pathology were showed below.<br>Which one is the MOST likely diagnosis for her?<br>A. Charcot-Marie-Tooth disease 1B<br>B. Charcot-Marie-Tooth disease II<br>C. Charcot-Marie-Tooth disease IA<br>D. Congenital insensitive to pain and anhidrosis<br>E. Charcot-Marie-Tooth disease X-linked
A-II	2018	15	A 20 year-old Taiwanese young man has suffered from frequent hyperpyrexia of unknown origin since childhood. He had a normal developmental milestone. He experienced frequent long bone fractures, dislocations, intractable osteomyelitis of right knee and traumatic amputation of toes. He also has mild

			mental retardation. His sural nerve pathology (shown below) revealed marked decreased small myelinated fibers and unmyelinated fibers, but normal large myelinated fibers. Which is the MOST likely diagnosis for him?<br>A. Amyloid neuropathy<br>B. Dejerine-Sottas disease<br>C. Roussy-Levy syndrome<br>D. Congenital insensitive to pain and anhidrosis<br>E. Riley Day syndrome
A-II	2018	17	A 60-year-old Taiwanese female with NIDDM suddenly developed an acute onset of the hemichorea-hemiballism (HB-HC) of the right extremities. There was neither a remarkable history of drug intake nor contributory past or family history. Neurological examination was normal, except for reduced deep tendon reflexes and right hemichorea-hemiballism.Unenhanced brain CT of this patient was shown below. Which is the MOST likely diagnosis for this patient?<br>A. Cerebral hemorrhage, left BG with HB-HC<br>B. Brain tumor, left BG with HB-HC<br>C. Cerebral infarction, left BG with HB-HC<br>D. Non-ketotic hyperglycemia with HB-HC<br>E. None of above
A-II	2019	15	A 70-year-old Taiwanese man with hypertension progressively developed slowness of the motion, unsteady gait and easily falling in past one year. The neurological examination revealed masked face, dysarthria, symmetrically bilateral bradykinesia, and loss of postural reflex, but there was no trembling movement at extremities. The rigidity was mild and symmetrical on his extremities. But he had marked rigidity on his neck. He was then treated by L-dopa under the impression of parkinsonism, but the drug seemed to be ineffective. T2-weighted MRI of brain is shown. Which one is the MOST likely diagnosis?<br>A. Parkinson’s disease (PD)<br>B. Spinocerebellar ataxia (SCA).<br>C. Progressive supranuclear palsy (PSP)<br>D. Multiple system atrophy-cerebellar type (MSA-C)<br>E. Corticobasal disease (CBD)
A-II	2021	25	30 years old Taiwanese man has had numbness and mild weakness of both feet for uncertain years. He is alcoholic addition. His elder brother has hand numbness as well. His nerve conduction study showed as below at his age of 17 years:<br>Motor conduction study<br>What is the most likely diagnosis?

			A), Alcoholic neuropathy<br>B), Vit B12 deficiency<br>C), Lead poison<br>D), Hereditary neuropathy with liability to pressure palsy<br>E), Charcot-Marie-Tooth disease 1B
A-II	2023	16	A 64-year-old male suffered from progressive weakness and paresthesia of four limbs for 2 years. Nerve conduction study revealed sensori-motor polyneuropathy with axonal involvement predominantly. The picture of sural nerve biopsy with Congo red stain in polarized light was shown below. His father and elder brother had a similar problem. Which statement is LEAST likely about the patient's clinical features?<br>A. Early autonomic dysfunction as orthostatic hypotension or frequent diarrhea.<br>B. Cardiomyopathy, heart failure or arrhythmias.<br>C. The most common mutation found in Taiwan is p. Ala117Ser.<br>D. Oral-form Tafamidis is a potent kinetic stabilizer of transthyretin.<br>E. Patisiran is one small interfering RNA (siRNA) to facilitate direct degradation of wild-type and mutant transthyretin protein.
A-II	2024	18	A 22 years old man presented with very slowly progressive symmetrical weakness of both lower limbs with distal muscular atrophy. His sural nerve biopsy and leg atrophy are shown below. Which of the following is FALSE?<br>A. Biopsy showed onion bulb formation, which is the result of repeated cycles of demyelination and remyelination<br>B. CMT1A, associated with PMP22 duplication, is the most common form of CMT in Taiwan<br>C. Transient CNS dysfunction and stroke-like episodes are reported in patients with CMT1X<br>D. The standard cutoff for demyelinating motor nerve conduction velocity is 38m/s in the tibial nerve<br>E. Currently there is no cure or effective disease-modifying treatment

Supplementary Figures

Figure S1. Full-stem screenshot used for DeepSeek input (question text + options + figure).

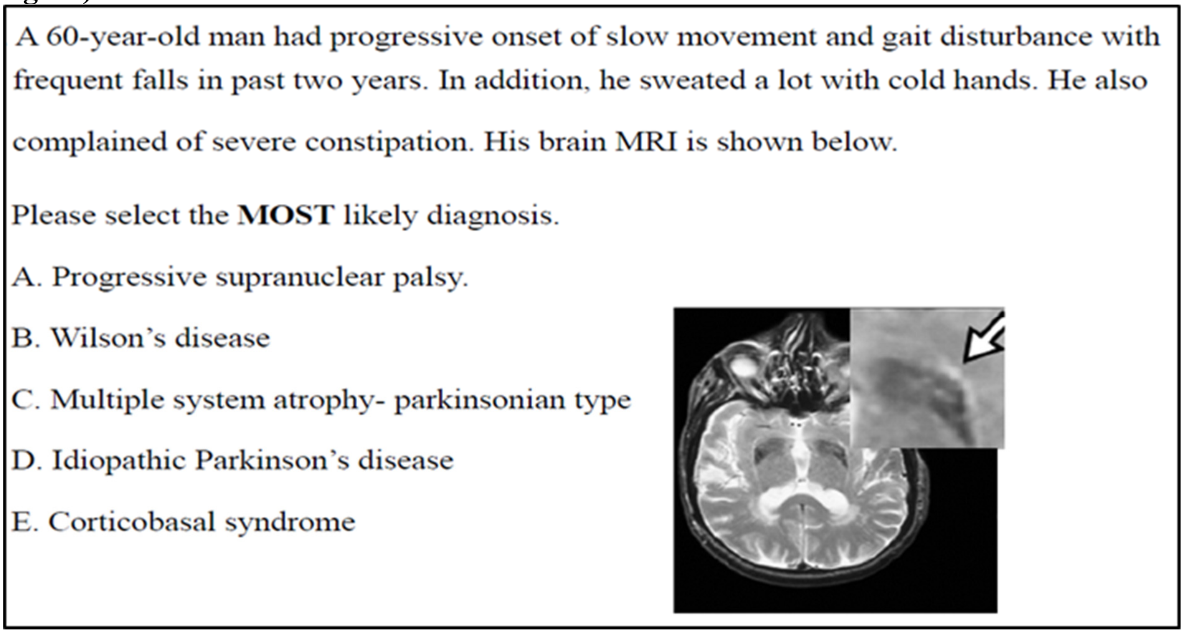

Example of a full-stem screenshot provided to DeepSeek-V3/R1 during evaluation. The screenshot contains the entire question stem, answer options, and the associated medical figure, ensuring that all textual information embedded in the item is available to the interface for processing.

Figure S2. DeepSeek response generated from a full-stem screenshot input.

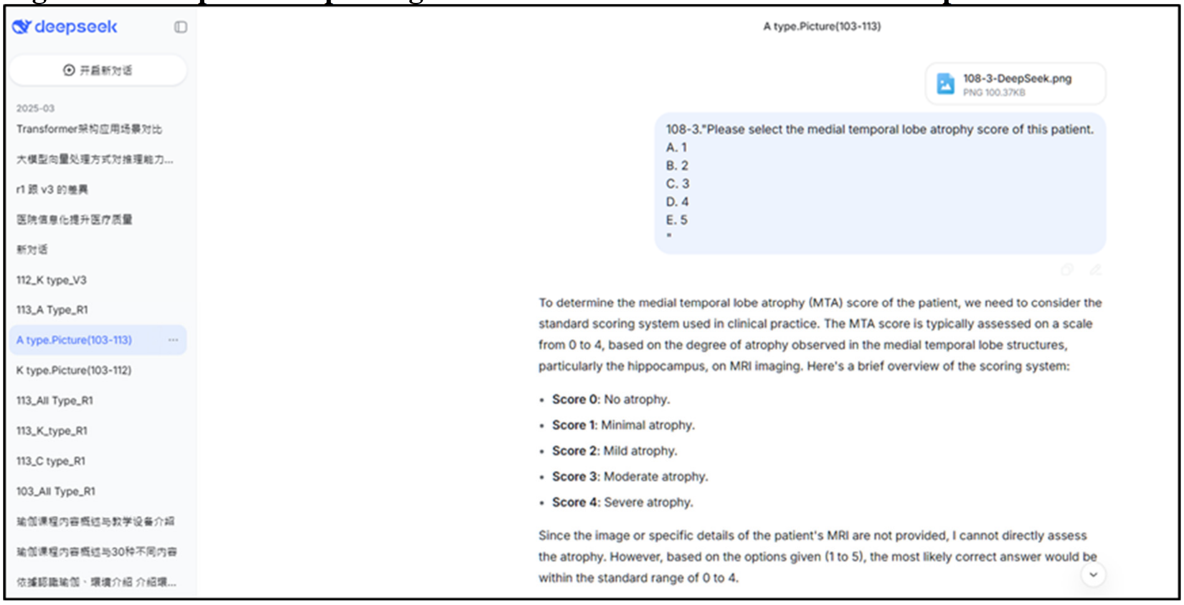

Representative DeepSeek output when prompted with a full-stem screenshot (Figure S1). This example illustrates that, under our evaluation interface, DeepSeek models could respond when the uploaded image contained readable embedded text (i.e., the item stem and options).

Figure S3. Image-only upload illustrating text-extraction failure in the DeepSeek interface.

(A)

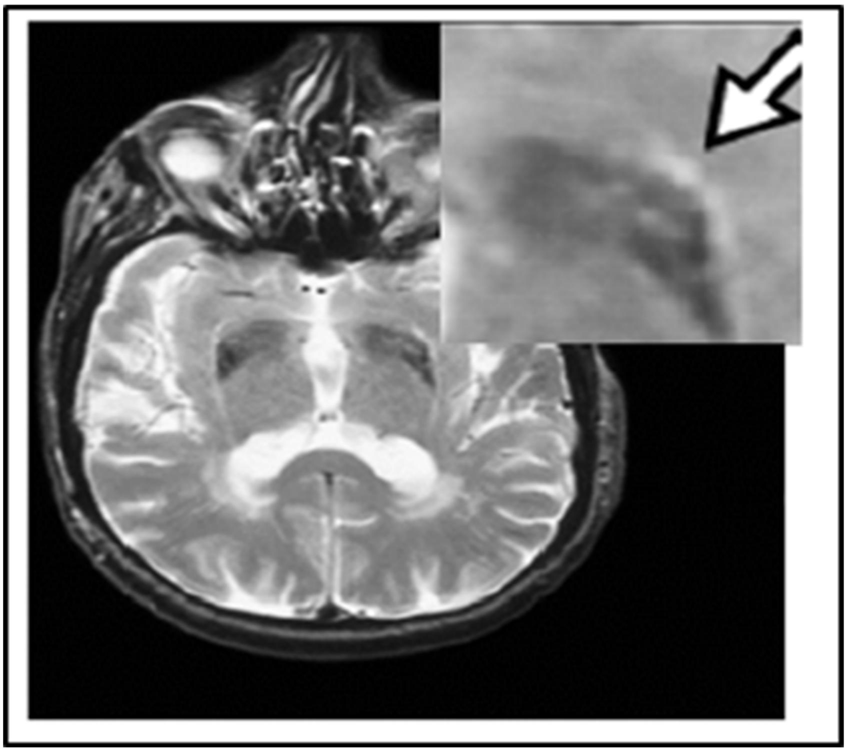

(B)

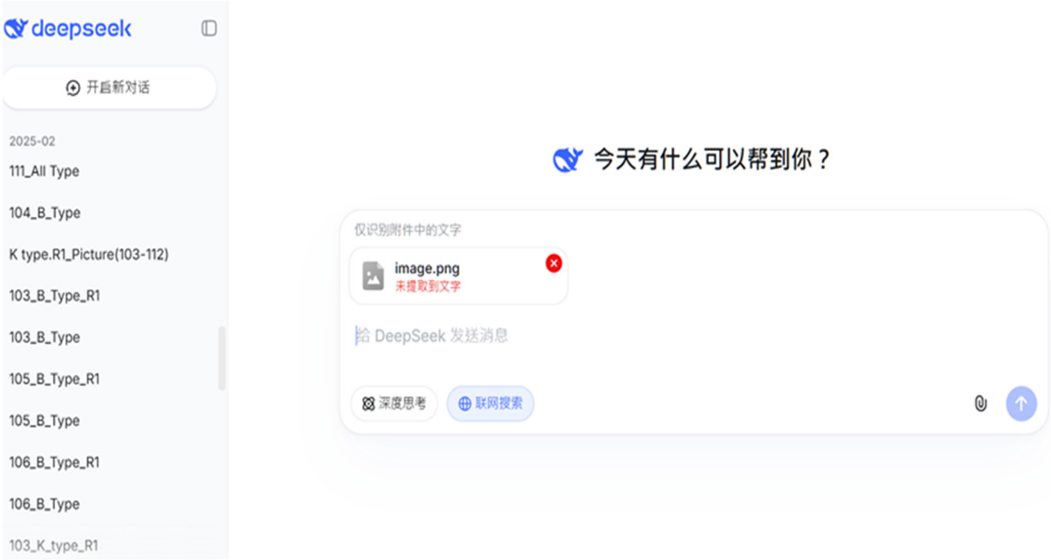

Illustration of the limitation observed in the DeepSeek public interface. (A) An image-only medical figure with minimal or no readable embedded text. (B) The interface message indicating that no text was extracted from the uploaded image ("text not extracted"), which prevented reliable processing. This constraint motivated our use of full-stem screenshots for A-II-type items.

Figure S4. Example of keyword-triggered refusal in DeepSeek-R1 during evaluation (prompt containing "Taiwan").

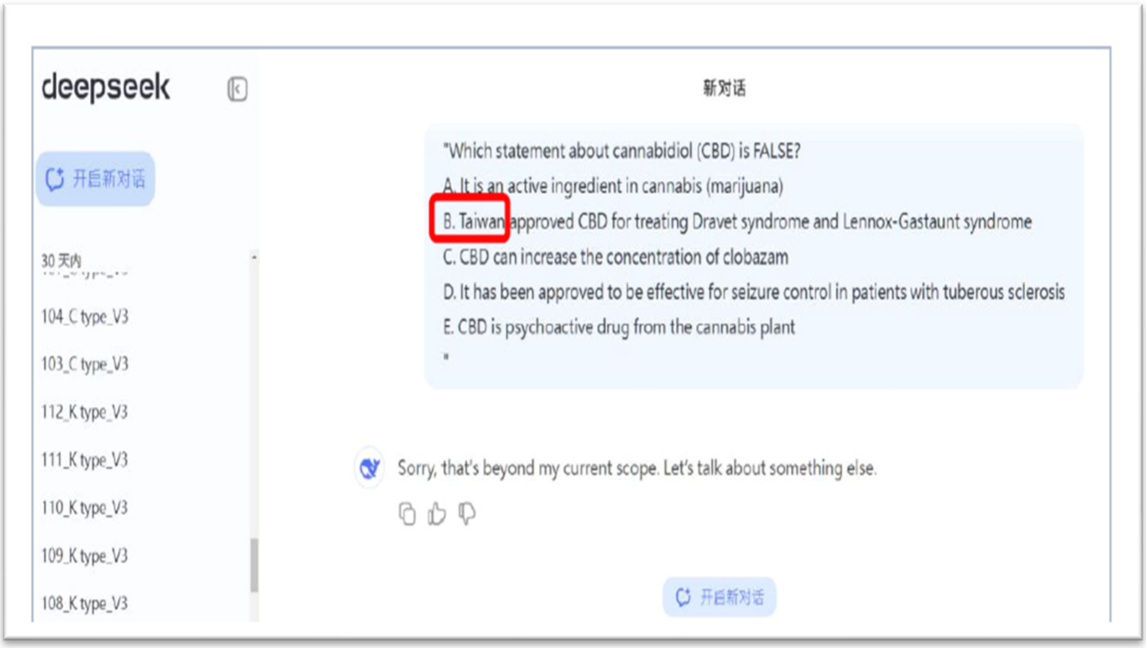

Figure S5. Summary of model accuracy by question type (2018–2024 average).

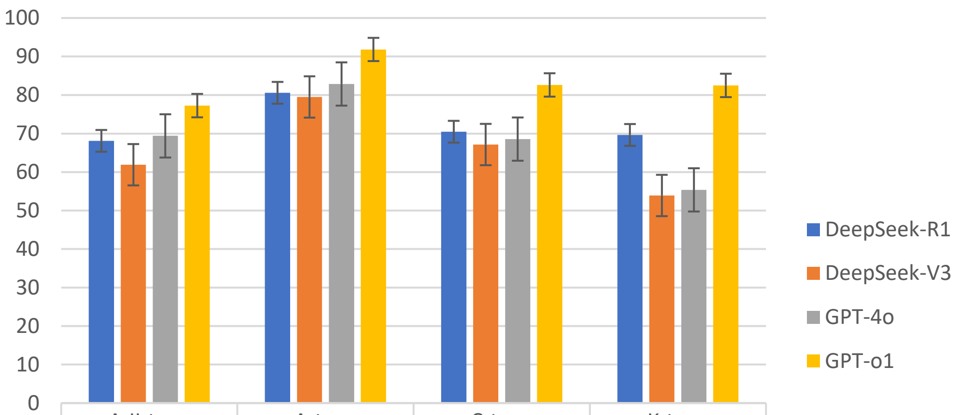

	A-II-type	A-type	C-type	K-type
DeepSeek-R1	68.1	80.59	70.48	69.64
DeepSeek-V3	61.9	79.51	67.14	53.93
GPT-4o	69.4	82.86	68.57	55.36
GPT-o1	77.26	91.84	82.62	82.5

Summary of model accuracy across question types. The figure summarizes the average accuracy (2018–2024) for each model by question format (A-, C-, K-, and A-II-type). Error bars indicate the standard error across years. Detailed year-by-year accuracies and overall averages are reported in Table S5.

Figure S6. Evaluation workflow and platform-specific operational constraints.

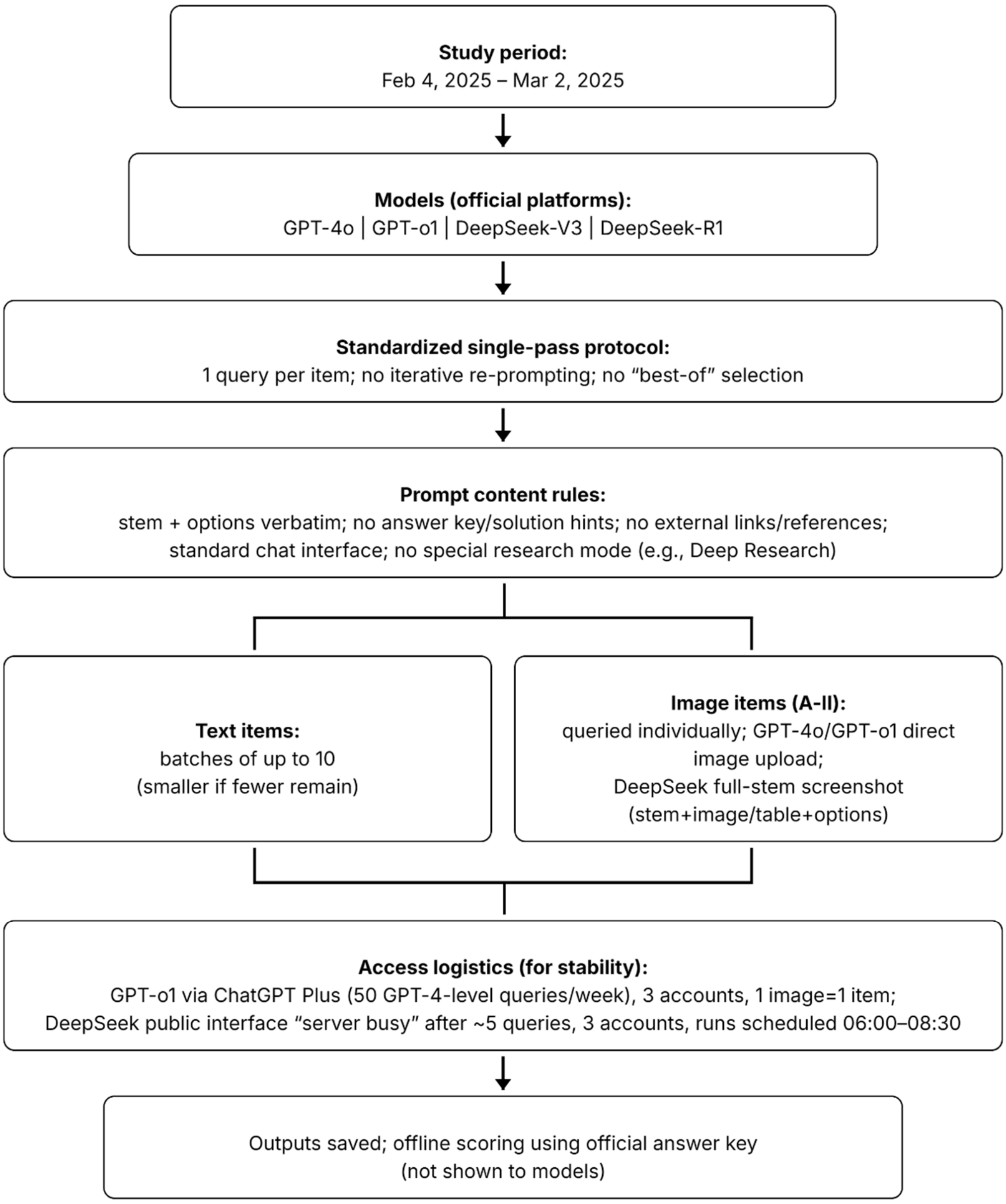

Supplement: Supplementary file 1 [file bioengineering-13-00302-s001.zip › bioengineering-4171823-supplementary.pdf]
