# Supplementary material for: Benchmarking Large Language Models on the Taiwan Neurology Board Examinations (2018–2024): A Comparative Evaluation of GPT-4o, GPT-o1, DeepSeek-V3, and DeepSeek-R1
Source: Bioengineering (Basel). 2026 Mar 5;13(3):302. doi: 10.3390/bioengineering13030302 (PMC13024452; doi:10.3390/bioengineering13030302)
Supplement: Supplementary file 1 [file bioengineering-13-00302-s001.zip › Supplementary_Methods.pdf]

Supplementary Methods

1. Access logistics and batching details

GPT-o1 was accessed via ChatGPT Plus accounts subject to weekly message limits (up to 50 GPT-4-level queries per week); three Plus accounts were used to complete the full single-pass evaluation. DeepSeek-V3 and DeepSeek-R1 were accessed via the public DeepSeek interface, where intermittent service interruptions (e.g., “The server is busy. Please try again later.”) were encountered; to maintain stability, we distributed queries across three accounts and scheduled runs during off-peak hours (6:00–8:30 AM). Due to these access constraints, the prompt sequence for DeepSeek-R1 occasionally differed from other models, but the total number of prompted items remained identical across all LLMs. For text-based questions, prompts were submitted in batches of up to 10 questions per input (smaller batches were used when fewer items remained), whereas image-based items were submitted individually. All items were evaluated under the standardized single-pass protocol (one query per item), and prompts did not include external links or references.

2. Standardized prompt templates and examples

To ensure comparability across platforms, we used fixed prompt templates across models. Templates below are provided in a copy/paste format. Bracketed text indicates where the exam stem/options should be inserted.

2.1 K-type (Multiple True-False; combination options A–E)

Instruction (copy/paste):

You must select one valid answer for each question; no question may be left unanswered or marked as invalid.
Answer the questions in order, providing a brief reason for each answer.
Then, create a table at the end listing only the answer choices for each original question number.

Questions: Multiple True-False
Select one of the following options:
(A) if 1, 2, and 3 are true
(B) if 1 and 3 are true
(C) if 2 and 4 are true
(D) if only 4 is true
(E) if all statements are true.

Every question must have a valid answer choice; responses such as 'No valid letter' are not allowed.

Questions:
[Paste the K-type questions here, in order.]

2.2 A-type (Single best answer; A–E)

Instruction (copy/paste):

Answer the questions in order, then create a table listing only the answer choices for each original question number at the end.

Questions:
[Paste the A-type questions here, each with options A–E.]

2.3 A-II-type (Image-based single best answer; A–E)

Instruction (copy/paste):

Read the attached image and select ONE appropriate answer (A, B, C, D, or E).

Input:
[Attach/upload the image (or full-stem screenshot).]

2.4 C-type (Two-statement format; A only / B only / Both / Neither)

Instruction (copy/paste):

Answer the questions in order, providing a brief reason for each answer.
Then, create a table at the end listing only the answer choices for each original question number.

For each question, select:
(A) A only
(B) B only
(C) Both A and B
(D) Neither A nor B.

Questions:
[Paste the C-type questions here, each containing statement A and statement B.]
